# Screening Oat Genotypes for Tolerance to Salinity and Alkalinity

**DOI:** 10.3389/fpls.2018.01302

**Published:** 2018-10-02

**Authors:** Jianhui Bai, Weikai Yan, Yuqing Wang, Qiang Yin, Jinghui Liu, Charlene Wight, Baoluo Ma

**Affiliations:** ^1^Ottawa Research and Development Centre, Agriculture and Agri-Food Canada, Ottawa, ON, Canada; ^2^Experimental Station of Agricultural Ministry for Eco-environment Observation of Sandy Grassland in Ordos, Institute of Grassland Research, Chinese Academy of Agricultural Sciences, Hohhot, China; ^3^Inner Mongolia Agricultural University, Hohhot, China

**Keywords:** salt tolerance, alkali tolerance, screening method, oat, GGE-biplot, cone-tainer

## Abstract

A set of four experiments was conducted to develop methods for screening oat tolerance to salt and alkali and the following results were obtained. (1) In experiment 1, 68.5 mmol L^-1^ salt and 22.5 mmol L^-1^ alkali were identified as appropriate concentrations for determining oat tolerance to salinity and alkalinity during germination. (2) These concentrations were used in experiment 2 to screen 248 oat genotypes and 21 were identified to be tolerant to salinity and alkalinity in germination. (3) In experiment 3, one salt treatment, 40 L of Na_2_SO_4_:NaCl (1:1), 150 mmol L^-1^, was found to be optimal for screening oat tolerance to salinity during growth and development. For alkalinity tolerance, the optimal treatment was 40 L of Na_2_CO_3_:NaHCO_3_ (1:1) at 75 mmol L^-1^. (4) No significant correlation was found between tolerances at the germination and adult stages or between tolerances to salt and alkali. Three lines were found to be tolerant to both salt and alkali in both germination and adult stages. (5) In experiment 4, 25 out of 262 oat genotypes were found to be tolerant to both salinity and alkalinity. (6) GGE biplot analysis was found to be effective in interpreting the multivariate data and the plastic cone-container system was found to be cost-effective system for screening adult plant tolerance to salt and alkali. (7) The symptoms of salt stress and alkali stress were found to be different; alkali stress mainly reduces the chlorophyll content, while salinity mainly disrupts water absorption.

## Introduction

Oats (*Avena sativa* L.) are grown throughout the world as grain, feed, forage, cover crops, and rotation crops. Oat-based food is considered healthy because of the high dietary fiber content of oat groats, particularly beta-glucan (Martínez-Villaluenga and Peñas, 2017). However, oat crops are less profitable than maize, soybean, or wheat crops. As a result, oats are usually grown in regions with short growing seasons (often at high latitudes or altitudes) or in problematic conditions such as drought, poor soil fertility, high salinity (high salt concentration with neutral pH), and high alkalinity (high salt concentration with high pH). Such soil and climatic conditions are less suitable for more profitable crops than they are for oats.

Salinity is a major abiotic stressor affecting crop production. More than 6% of the world’s total land area is affected by salinity (Gao et al., 2016). All of these problematic soils have the potential to be used for growing oat crops.

Oat crops are considered to be moderately tolerant to salinity and alkalinity. Field studies in northeast China have shown that some oat cultivars are able to grow in soil with pH values as high as 9.0 (Zhao et al., 2007; Bai et al., 2013). High oat yields can be achieved in saline and alkaline soils if irrigation is provided, which is the current practice in some regions of Jilin Province, China. Soil salinization always occurs in arid areas. However, irrigation is usually not an option in arid and semi-arid regions or in regions where it is not economically viable or sustainable. A sustainable solution is to develop oat cultivars that are tolerant to salinity and alkalinity. Studies on the tolerance of oat crops to salinity and alkalinity are rare and, to date, only a few have been published (Oraby et al., 2005; Zhao et al., 2007; Oraby and Ahmad, 2012). Therefore, there is a need for a better understanding of the genetic variations affecting saline/alkaline tolerance in a large population of oat genotypes, and a method of screening for these.

However, the successful application of such methods to the breeding of salt-tolerant oats has been limited by many factors, such as the lack of a standard, effective method for evaluating salt tolerance (Talei et al., 2013). In this study, we improved the efficiency of screening methods using three approaches: (1) increasing the number of cultivars, (2) finding an appropriate salt treatment scheme that provides optimal discriminating power in tests of salt tolerance (the suitable concentration and volume of salt solution), and (3) determining suitable criteria for salt tolerance.

Most previous studies on salt tolerance mechanisms are based on a rather limited number of genotypes, which is clearly insufficient to convince plant breeders that certain traits can be used as key selection criteria for salt tolerance (Zhu et al., 2016). Recently, a new and simple screening method called the plastic “cone-tainer” method was proposed. This method is less laborious and time consuming than previously reported methods. It involves a scheme where salt tolerance can be evaluated among 48 genotypes within a space of 2 m^2^, enabling testing of a greater number of genotypes. The effects of salt stress on soybeans have been well documented using this method (Lee et al., 2008). However, plastic cone-tainers have not been used to screen for salt-tolerant genotypes in oats.

Suitable salt treatments are essential for increasing screening efficiency. Salt concentrations that are too low cannot demonstrate genetic differences, and screening for the survival of different varieties under high salinity stress may be unproductive. The *discriminating power* function of the GGE biplot software can analyze multiple salt treatments and determine the one with the highest discriminating power. However, this method has not yet been used to identify salt-tolerant genotypes (Yan and Kang, 2002; Munns et al., 2006; El-Hendawy et al., 2009; Yan, 2014; Yan et al., 2017).

Some studies have reported that investigation of a plant’s germination stage allows good prediction of its response to salinity (Wang et al., 2011). However, others have reported that long-term experiments allow more reliable salt tolerance screening (Zhu et al., 2016). Thus, to address this uncertainty, this study used both germination rate and yield per plant to measure salinity tolerance.

Both neutral (pH = 7) and alkaline (pH > 7) salts exist in soil. It is now accepted that alkalinity affects plants more than salinity does (Zhang et al., 2013; Zhu et al., 2016). However, previous studies have mostly concentrated on neutral salt stress (Munns and James, 2003; Lee et al., 2005; Genc et al., 2007; Faiyue et al., 2012; Talei et al., 2013; Sakina et al., 2016), while alkaline stress has received little attention.

The purposes of this study were to: (1) identify salt and alkali concentrations and procedures that can be used to effectively screen for oat tolerance during seed germination and/or plant growth. (2) Identify oat genotypes, from a large population, that are tolerant to salinity and/or alkalinity during seed germination and plant growth; and (3) study the relationship between tolerance to salinity and tolerance to alkalinity during seed germination, plant growth, and development.

## Materials and Methods

This study was conducted in a greenhouse at the Ottawa Research and Development Center of Agriculture and Agri-Food Canada in Ottawa, Ontario, during 2015 and 2016. The study included a series of four experiments, which are described below.

### Experiment 1

To determine the median lethal concentration of salt (or alkali), seeds of four oat cultivars adapted to eastern Canada, namely, AAC Bullet, AC Dieter, AC Bradley, and AAC Nicolas, were germinated in a hydroponic nutrient solution with various salinity and alkalinity levels. Eight saline solutions were created using 10 mL Hoagland solution in petri dishes containing 0, 15, 30, 45, 60, 75, 90, or 105 mmol L^-1^ salt (NaCl:Na_2_SO_4_ = 1:1; Sigma–Aldrich Company, St. Louis, MO, United States). Nine alkaline solutions were created using 10 mL Hoagland solution in petri dishes containing 0, 4, 8, 12, 16, 20, 24, 28, or 32 mmol L^-1^ alkali (Na_2_CO_3_:NaHCO_3_ = 1:1; Sigma–Aldrich Company, St. Louis, MO, United States). In each petri dish, 50 seeds were placed on double-layer filter paper. Each treatment condition was performed in triplicate. The petri dishes were sealed with parafilm to prevent water loss and were placed in an incubator with a 24 h dark regime at 23°C and 80% relative humidity for 7 days. A seed was considered to have germinated when its embryonic bud reached half of its length.

A germination rate index (GRI) was calculated using the following formula:

GRI =x/y,

where *x* = number of seeds germinated after 7 days and *y* = number of seeds per petri dish (50).

The median lethal concentrations of salt (or alkali) (LC50: concentration at which the germination of 50% of the seeds was inhibited by stress) were calculated from the germination rates under varied salinity (or alkalinity) levels using regression analysis.

### Experiment 2

To determine the tolerant and sensitive oat genotypes during the germination stage, 248 oat genotypes (**Supplementary Table 1**) were used. These genotypes were tested for germination under three treatments: (1) control: 10 mL Hoagland solution in a petri dish without adding any salt or alkali, (2) alkali treatment: 10 mL Hoagland solution in a petri dish with median lethal concentration of alkali (Na_2_CO_3_:NaHCO_3_), (3) salt treatment: 10 mL Hoagland solution in a petri dish with median lethal concentration of salt (NaCl:Na_2_SO_4_). The median lethal concentration of salt (or alkali) was from the results of experiment 1. According to the data on the germination rates of the 248 oat genotypes under the three treatments, the salt (or alkali) tolerant genotypes could be screened during the germination stage. The experimental procedures used to determine the GRI were the same as those used in experiment 1.

### Experiment 3

The next step was to determine whether the oat genotypes that demonstrated salt or alkali tolerance during germination were also tolerant during the growth and development stage. Accordingly, 43 oat genotypes that were considered tolerant or sensitive to salt and alkali stress during germination were grown in a greenhouse to test their tolerance during growth and development.

In Experiment 3, to identify the salt (or alkali) treatment that provided the highest discriminating power for tolerance during growth and development, 19 salt and alkali treatments were used, as described in **Table 1**.

**Table 1:** List of treatments used in the greenhouse study (Experiment 3).

Treatment ID	Name	Implementation
1	Control	Control (Hoagland solution)
2	Saline 2	Na_2_SO_4_:NaCl = 1:1, 100 mmol L^-1^, 32 L
3	Saline 3	Na_2_SO_4_:NaCl = 1:1, 100 mmol L^-1^, 40 L
4	Saline 4	Na_2_SO_4_:NaCl = 1:1, 100 mmol L^-1^, 48 L
5	Saline 5	Na_2_SO_4_:NaCl = 1:1, 150 mmol L^-1^, 32 L
6	Saline 6	Na_2_SO_4_:NaCl = 1:1, 150 mmol L^-1^, 40 L
7	Saline 7	Na_2_SO_4_:NaCl = 1:1, 150 mmol L^-1^, 48 L
8	Saline 8	Na_2_SO_4_:NaCl = 1:1, 200 mmol L^-1^, 32 L
9	Saline 9	Na_2_SO_4_:NaCl = 1:1, 200 mmol L^-1^, 40 L
10	Saline 10	Na_2_SO_4_:NaCl = 1:1, 200 mmol L^-1^, 48 L
11	Alkaline 11	Na_2_CO_3_:NaHCO_3_ = 1:1, 50 mmol L^-1^, 32 L
12	Alkaline 12	Na_2_CO_3_:NaHCO_3_ = 1:1, 50 mmol L^-1^, 40 L
13	Alkaline 13	Na_2_CO_3_:NaHCO_3_ = 1:1, 50 mmol L^-1^, 48 L
14	Alkaline 14	Na_2_CO_3_:NaHCO_3_ = 1:1, 75 mmol L^-1^, 32 L
15	Alkaline 15	Na_2_CO_3_:NaHCO_3_ = 1:1, 75 mmol L^-1^, 40 L
16	Alkaline 16	Na_2_CO_3_:NaHCO_3_ = 1:1, 75 mmol L^-1^, 48 L
17	Alkaline 17	Na_2_CO_3_:NaHCO_3_ = 1:1, 100 mmol L^-1^, 32 L
18	Alkaline 18	Na_2_CO_3_:NaHCO_3_ = 1:1, 100 mmol L^-1^, 40 L
19	Alkaline 19	Na_2_CO_3_:NaHCO_3_ = 1:1, 100 mmol L^-1^,48 L


**FIGURE 1:**
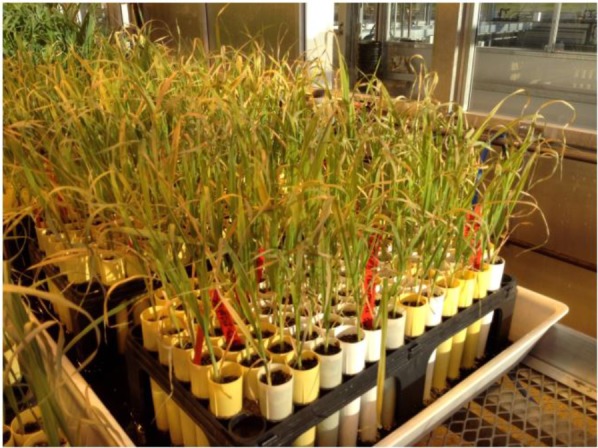
The cone-tainer planting system.

The 43 oat genotypes were planted separately in cone-tainers (**Figure 1**), each filled with approximately 160 cm^3^ of vermiculite. Each 120 L sterilite container can accommodate three racks, and each rack can hold 98 plastic cones that are 15 cm tall and 3.5 cm in diameter at the top (Stuewe & Sons Inc., Tangent, OR, United States).

Each genotype was seeded into six cones (six replicates) within each rack. Thus, six cone-tainers per genotype were used for screening (**Figure 1**), and 16 genotypes were placed in a cone-tainer rack for evaluation. Each cone was seeded with three seeds and thinned to a single plant at the two-leaf stage. We used tap water with Hoagland solution to keep the plants moist.

Each cone had two holes at the bottom, allowing the plants to take up water and nutrients from the sterilite container. When the third leaf was fully developed, salt or alkali solution was added to the bottom of the sterilite container for the plants to take up. The stress treatments were created by adding different volumes and concentrations of salt or alkali in the sterilite container, similar to those used in Ledesma et al. (2016). Each sterilite container represented a treatment, and each cone represented a replication within the treatment. A total of 19 treatments were created, including 9 concentrations of salinity, 9 concentrations of alkalinity, and a control treatment without any salt or alkali (**Table 1**).

The number of seeds per plant (43 genotypes under 19 treatments) was determined at the termination of the experiment. The data were analyzed using the GGE biplot software to determine the salt or alkali concentration that provided the best discrimination of the genotypes.

To determine the various effects of salt and alkali stresses on oats, and to determine the physiological differences between tolerant and sensitive genotypes, the chlorophyll content, number of yellow leaves, number of dry leaves at the heading stage, number of tillers, and number of panicles were determined for each plant.

### Experiment 4

To validate the screening method established from Experiment 3, another 262 oat genotypes were used (**Supplementary Table 2**). These 262 oat genotypes were provided by four breeding institutions: the University of Saskatchewan, North Dakota State University, and the Brandon and Ottawa Research and Development Centres of Agriculture and Agri-Food Canada. Therefore, they represent a wide genetic basis, diversity, and adaptability.

### Data Analysis

Conventional statistical analyses and biplot analysis were conducted using GGE biplot software (Yan et al., 2017). Biplot analysis was developed according to Gabriel (1971) and popularized in analysis of agricultural and life-science data following Yan et al. (2000) and Yan and Kang (2002). GGE stands for genotypic main effect plus genotype by environment interaction. GGE biplot analysis consists of a set of graphs that allow visualization of the patterns in a dataset from different angles (Yan and Kang, 2002; Yan and Tinker, 2006; Yan et al., 2017). GGE biplot analysis has been mainly used to analyze data from multi-environment variety trials, but it is equally useful for analyzing other types of data that can be organized a two-way table.

## Results

### Determining Optimal Saline and Alkaline Concentrations for Screening the Tolerance of Oats During Germination

To select an appropriate salt or alkali concentration for screening the tolerance of oat plants during germination, we first investigated the effects of nine salt and alkali concentrations on germination rates. As shown in **Tables 2** and **3**, the number of germinated seeds decreased as the salinity and alkalinity concentrations increased. However, this decrease was not statistically significant until the concentrations reached 60 mmol L^-1^ salt and 16 mmol L^-1^ alkali (**Table 2**). Thereafter, germination was increasingly affected as the alkali and salt concentration increased further (**Table 3**). These results indicate that salt solutions used for screening germination tolerance should have concentrations exceeding 60 mmol L^-1^, while alkali concentrations should exceed 16 mmol L^-1^.

**Table 2:** Number of germinated seeds (out of 50) for four oat genotypes under different concentrations of salt.

Salinity levels (mmol L^-1^)	AC Bradley	AAC Bullet	AC Dieter	AAC Nicolas	Mean
0	43.3	33.3	35.3	42.3	38.6
15	32.7	29.7	25.3	29.3	29.3
30	37.0	24.7	33.3	36.7	32.9
45	41.0	38.0	28.0	35.3	35.6
60	39.3	27.7	10.3	36.7	28.5
75	40.3	23.7	0.7	24.3	22.3
90	13.7	1.3	0.0	3.7	4.7
105	5.3	0.0	0.0	0.7	1.5
*N*	3	3	3	3	12
LSD (*P* = 0.05)	8.1	10.4	8.5	7.0	7.3


**Table 3:** Number of germinated seeds (out of 50) for four oat genotypes under different concentrations of alkali.

Alkaline levels (mmol L^-1^)	AC Bradley	AAC Bullet	AC Dieter	AAC Nicolas	Mean
0	43.3	33.3	35.3	42.3	38.6
4	47.7	42.7	38.0	36.3	41.2
8	44.0	39.0	30.3	42.7	39.0
12	47.7	38.7	25.0	33.7	36.3
16	42.7	24.0	30.7	30.3	31.9
20	37.3	19.3	20.7	28.0	26.3
24	29.0	26.0	17.0	20.7	23.2
28	28.7	14.0	7.3	21.0	17.8
32	12.7	1.7	1.0	1.0	4.1
*N*	3	3	3	3	12
LSD (*P* = 0.05)	9.7	8.9	10.2	10.4	6.1


**FIGURE 2:**
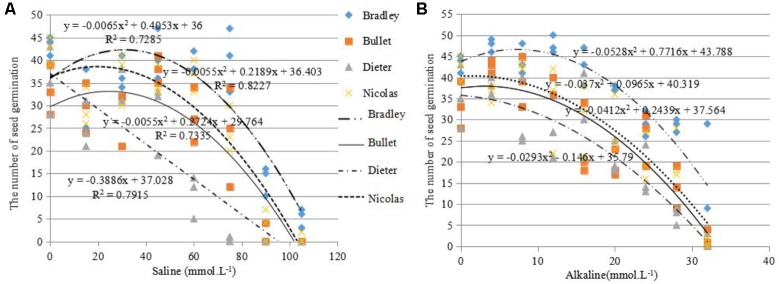
Number of seeds germinated (out of 50) under different levels of salinity **(A)** and alkalinity **(B)** for four different oat cultivars: AC Bradley, AAC Bullet, AC Dieter, AAC Nicolas.

For this reason, we used the salt and alkali LC50s (concentrations at which 50% of seeds fail to germinate) to screen for germination tolerance. The average salt LC50 value for the four genotypes was 68.5 mmol L^-1^ (>60 mmol L^-1^), the average alkali LC50 value for the four genotypes was 22.5 mmol L^-1^ (>16 mmol L^-1^; **Figure 2**).

### Screening of 248 Oat Genotypes for Salinity and Alkalinity Tolerance During the Germination Stage

To identify tolerant and sensitive genotypes during germination, we observed the germination rates of 248 oat genotypes subjected to salt LC50 (68.5 mmol L^-1^) and alkali LC50 (22.5 mmol L^-1^) treatments. The data were analyzed by GGE biplot.

There were genotypic differences in salinity and alkalinity tolerance during germination of the 248 genotypes. Some of the results from GGE biplot are shown in **Tables 4** and **5**. The 21 oat genotypes with the highest germination rates under saline and alkaline conditions (i.e., tolerant) are listed in **Table 4**. Their IDs are 126, 125, 140, 80, 105, 118, 6, 112, 127, 119, 128, 121, 108, 4, 132, 115, 165, 214, 133, 171, and 16. The 22 oat genotypes with lowest germination rates (i.e., sensitive) are listed in **Table 5**, and were 153, 183, 5, 176, 184, 83, 25, 137, 233, 76, 79, 8, 227, 11, 21, 67, 219, 170, 168, 66, 240, and 155.

**Table 4:** The top 21 genotypes out of 248 for germination rates under salt and alkali stresses.

Variety	Mean	Mean/LSD5%	Class5%	Variety	Mean	Mean/LSD5%	Class5%
126 Oa1434-1	1.85	45.55	A	121 Oa1432-5	0.75	18.44	
125 Oa1433-1	1.83	45.07	A	108Oa1426-7	0.69	17.08	
140Nd120609	1.73	42.65		4 Sa120091	0.66	16.16	
80 Oa1414-3	1.60	39.29		132Oa1438-1	0.61	15.04	
105Oa1426-4	1.48	36.43		115Oa1430-1	0.56	13.87	
118Oa1432-2	1.30	31.97		165Nd122569	0.45	11.15	
6 Sa120097	1.19	29.36		21409p05-dg	0.34	8.28	
112Oa1429-1	1.09	26.83		133Nd120042	0.29	7.20	
127 Oa1435-1	0.88	21.65		171Nd120580	0.02	0.51	
119Oa1432-3	0.88	21.54		16 Sa120850	0.016	0.39	
128 Oa1435-2	0.82	20.12					


*The genotypes can be compared for their “mean/LSD5%”. Tow genotypes are significantly different at *P* < 0.05 if they differ by > 1.0. A: not significantly different from the best at *P* = 0.05 level.*

**Table 5:** The poorest 22 genotypes in germination under salt and alkali stresses.

Variety	Mean	Mean/LSD5%	Class5%	Variety	Mean	Mean/LSD5%	Class5%
153Nd121147	-0.15	-3.59		8Sa120745	-0.84	-20.69	
183Nd121722	-0.16	-3.81		22709p09-ec	-0.85	-20.88	
5 Sa120093	-0.426	-10.41		11Sa120826	-0.90	-22.09	
176Nd121383	-0.486	-11.83		21809p06-eh	-0.94	-23.06	
184Nd121726	-0.59	-14.45		67Oa1410-1	-0.99	-24.35	
83Oa1414-6	-0.65	-15.94		21909p06-es	-1.03	-25.24	
25Sa120161	-0.67	-16.39		170Nd120497	-1.03	-25.3	
137Nd120430	-0.78	-18.64		168Nd120494	-1.09	-26.89	
23309p09-fa	-0.788	-19.22		66Sa110522	-1.29	-31.74	
76Oa1413-7	-0.798	-19.44		240 09p09-gp	-1.55	-37.99	
79Oa1414-2	-0.82	-20.06		155Nd121165	-1.65	-40.57	


The *mean* column contains the mean germination rates under saline and alkaline treatments for each genotype, based on centered and scaled data. The 21 tolerant genotypes showed significantly better tolerance than the 22 sensitive genotypes, with the average germination rate ranging from 0.016 to 1.85 for the tolerant genotypes and from -0.5 to -1.65 for the sensitive genotypes. These results indicate that the salt and alkali LC50s can be used to screen for tolerance during germination, and that GGE biplot analysis is effective in distinguish between tolerant and sensitive genotypes.

**FIGURE 3:**
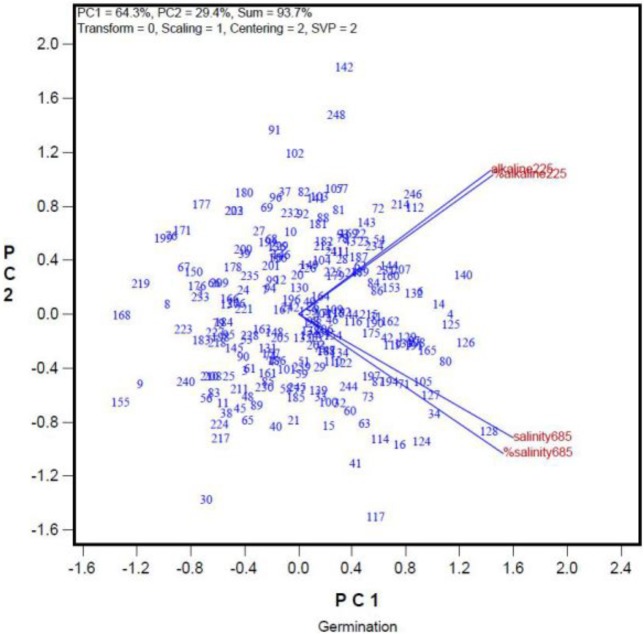
Relationship among germination rates under a salt stress and alkali stress across genotypes. The germination rate was represented both in number of germinated seed and in percentage of the control.

In addition, we used GGE biplot to investigate whether seed quality can affect germination rates under saline and alkaline stresses. The germination rates of the 248 oat genotypes under saline and alkaline treatments are summarized in the GGE biplot in **Figure 3**. The genotypes are represented by their ID (For their full names see **Supplementary Table 1**). “Salinity685” denotes the number of seeds that germinated in the 68.5 mmol L^-1^ salt treatment, and “%Salinity685” denotes the percentage of seeds germinated in the 68.5 mmol L^-1^ salt treatment relative to that in the control. Similarly, “Alkalinity225” refers to the number of seeds germinated in the 22.5 mmol L^-1^ alkali treatment, and “%Alkalinity225” refers to the percentage of seeds that germinated in the 22.5 mmol L^-1^ alkali treatment relative to that in the control. The cosine of the angle between two indexes can be used to approximate a Pearson correlation between them. Thus, “Salinity685” was closely correlated with “%Salinity685.” “Alkalinity225” was positively correlated with “%Alkalinity225,” as indicated by the acute angle between them (**Figure 3**). This indicates that the trend remained the same when the data were represented as the number of germinated seeds, or as a percentage relative to controls (**Figure 3**), despite variations in the germination rates of the genotypes in the control treatment. Thus, in this study, we used the number of seeds that germinated in the salt and alkali treatments, and did not consider the effects of seed quality on germination rates. This simplified the experimental procedure. More importantly, **Figure 3** showed there was little correlation between germination under the salt treatment and that under the alkali treatment, as indicated by the near-right angle between them.

### Determining Saline and Alkaline Treatments to Screen for Tolerance at the Growth and Development Stage

The relationship between tolerance at the germination and growth stages remains unclear. To assess whether salt or alkali tolerance during germination stage was correlated with tolerance during growth and development stage, a total of 43 genotypes identified as tolerant or sensitive during germination (**Tables 4** and **5**) were used in this experiment (experiment 3).

**FIGURE 4:**
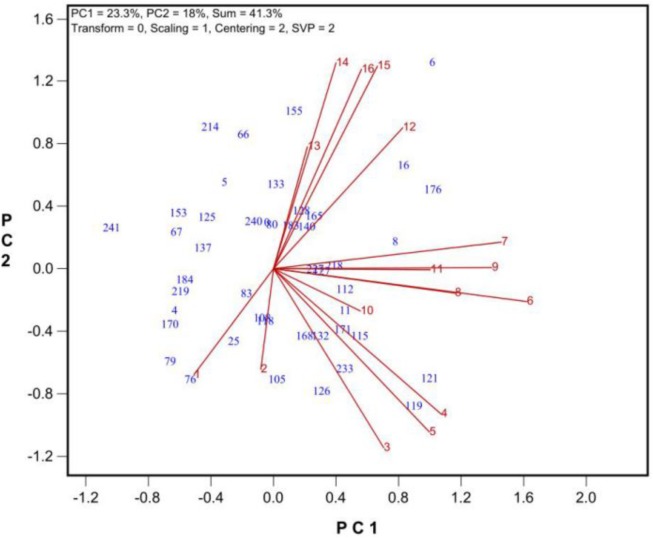
The similarity/dissimilarity in genotypic diffidences in grain set under 9 levels of salt stress (treatments 2 to 10) and 6 levels of alkali stress (treatment 11 to 16) plus the control (treatment 1). See **Table 2** for descriptions of the treatments.

Among the traits were determined, the number of grains per plant was the most integrative measure of tolerance during growth and development (**Figure 4**). Thus, the number of grains produced per plant will hereafter be used as a measure of tolerance during growth and development.

To find the best salt and alkali treatments to screen for oat tolerance at the growth and development stage, GGE biplot analysis was used. In Experiment 3, 43 oat genotypes were grown under 19 saline and alkaline treatments (9 levels of salinity from 2 to 10, 9 levels of alkalinity from 11 to 19, plus 1 control, **Table 1**). The biplot in **Figure 4** summarizes the number of grains produced under the 19 saline and alkaline treatments for the 43 genotypes in Experiment 3.

In **Figure 4**, the biplot is based on data centered (“Centering = 2”) and standard deviation-scaled (“Scaling = 1”) by the treatment, with singular values portioned into the treatment vectors (“SVP = 2”). **Figure 4** reveals the following patterns. First, among the salinity levels, treatment S6 was the most representative and discriminative, and among the alkalinity levels, treatment A15 was the most representative and discriminative, as indicated by their vector length and their angles with other treatments. Hence, these two treatments should be used to screen oat genotypes for tolerance to salinity and alkalinity during the growth and development stage. Little varietal differences were detected in treatments that had short vectors, such as treatments 2 and 13. Second, the genotypes responded differently to salinity and alkalinity, as indicated by the near right angles between the salinity levels (3, 4, 5, 6, 7, 8, 9) and alkalinity levels (12, 13, 14, 15, 16). This result suggests that there are different mechanisms controlling tolerance to salinity and alkalinity during the growth stage.

### Establishment of the Screening Method

We further examined the correlation between salt and alkali tolerance during the germination stage and growth stage. As shown in **Table 6**, tolerances during seed germination stage as represented by germination rate under the 22.5 mmol L^-1^ alkalinity treatment (“Germination_A22.5”) and under the 68.5 mmol L^-1^ salinity treatment (“Germination_S68.5”) were not correlated with tolerance during growth stage as represented by grain number produced under the S6 salinity treatment (“Seed_Saline6”) and under the A15 alkalinity treatment (“Seed_Alkaline15”). This result suggests that the genetic control and physiological mechanisms of tolerance are different in the germination and growth stages. For example, genotype 4SA120091 had a high germination rate but did not produce many seeds under salt and alkali stresses (**Supplementary Table 3**).

**Table 6:** Non-significant correlations among tolerances to salt and alkali in seed germination and seed-set across 43 genotypes.

	Seed_Saline6	Seed_Alkaline15	Germination_S68.5	Germination_A22.5
Seed_Saline6	1	0.073	0.158	0.071
Seed_Alkaline15	0.073	1	0.014	-0.011
Germination_S68.5	0.158	0.014	1	0.304
Germination_A22.5	0.071	-0.011	0.304	1


*Germination_S68.5, germination rate under a salinity treatment (68.5 mmol L^-*1*^); Germination_A22.5, germination rate under a alkalinity treatment (22.5 mmol L^-*1*^); Seed_Saline6, grain number under a salinity treatment (Saline6); Seed_Alkaline15, grain number under a alkalinity treatment (Alkaline15). Combined results from experiments 2 and 3.*

Our results indicate that it is essential to consider both germination and growth stages to establish effective greenhouse screening methods. Thus, with this screening technique, we screened oat genotypes at the germination stage under 68.5 mmol L^-1^ salt stress and 22.5 mmol L^-1^ alkali stress. Then, we screened another set of genotypes at the growth and development stage in plastic cone-tainers under S6 and A15 treatments (**Table 1**).

### Screening of 262 Oat Genotypes for Salinity and Alkalinity Tolerance With the Proposed Method

**FIGURE 5:**
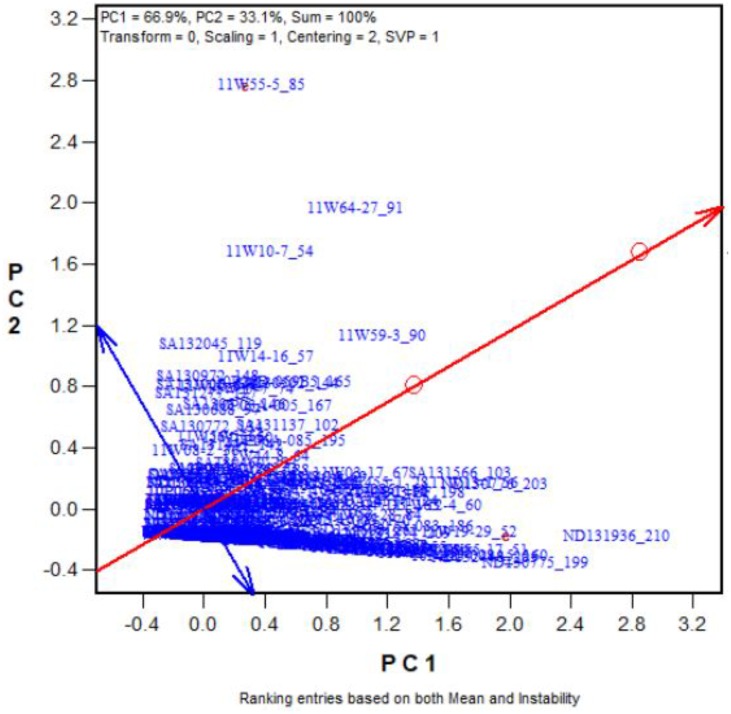
Ranking the 262 genotypes based on their tolerance (in terms of number of grains produced) across the salt and alkali stresses.

To validate our proposed salt and alkali tolerance screening method, another 262 oat genotypes were selected. **Figure 5** shows the “Rank entries” view of the biplot. The genotypes are ranked along the line with a single arrow, which points to better tolerance. The circle indicates the optimum genotype with the highest yield under the two stresses. The tolerant genotypes that had high numbers of seeds under the two stresses are placed near the circle. For example, Entry 210 (ND131936) stood out as the most tolerant genotype (also see **Supplementary Tables 4**, **5**). Genotype 199ND130775 was also tolerant, while 260ND132528 was a sensitive genotype. Among the 262 oat genotypes, the top 25 with the highest tolerances to salt and alkali stresses are listed in **Supplementary Tables 4**, **5**. These results indicate that the proposed screening method is effective.

### Relationships Between Tolerance to Salinity and Alkalinity

To assess whether alkalinity and salinity have different effects on oats, the grain numbers and some physiological indices obtained under salt stress were compared to those obtained under alkali stress. **Table 6** shows that the number of grains produced under salt stress was not correlated with that produced under alkali stress. The grain numbers of the 43 oat genotypes subjected to the 75 mmol L^-1^ alkali treatment were lower than those subjected to the 150 mmol L^-1^ salt treatment (**Supplementary Table 3**). These results indicate that alkalinity inhibits yield more severely than salinity.

**FIGURE 6:**
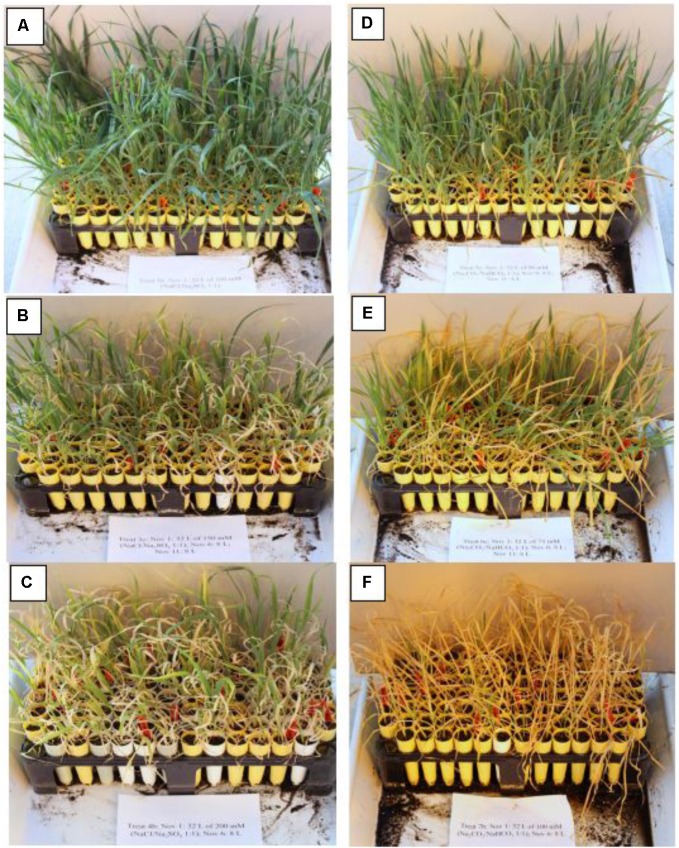
Appearance of oat plants under salt and alkali stresses. **(A–C)** salt stress. **(D–F)** alkali stress. **(A)** Oat under l00 mmol L^-1^ salt stress. **(B)** Oat under 150 mmol L^-1^ salt stress. **(C)** Oat under 200 mmol L^-1^ salt stress. **(D)** Oat under 50 mmol L^-1^ alkali stress. **(E)** Oat under 75 mmol L^-1^ alkali stress. **(F)** Oat under l00 mmol L^-1^ alkali stress.

**FIGURE 7:**
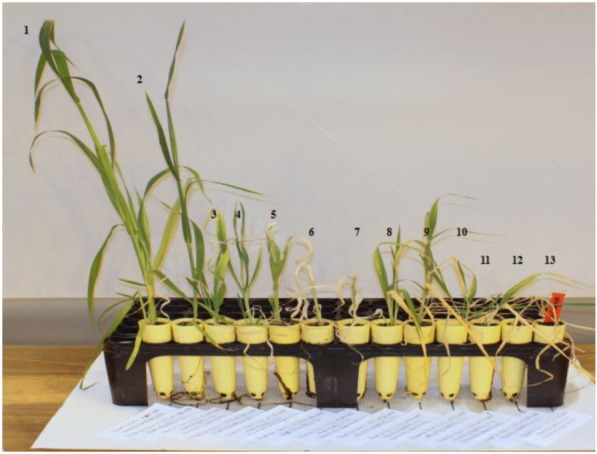
Plant height of a single genotype under varied salt and alkali stresses. 2–7, salt stress; 8–13, alkali stress. 1, Control; 2 and 3, oat under l00 mmol L^-1^ salt stress; 4 and 5, oat under 150 mmol L^-1^ salt stress; 6 and 7, oat under 200 mmol L^-1^ salt stress; 8 and 9, oat under 50 mmol L^-1^ alkali stress; 10 and 11, oat under 75 mmol L^-1^ alkali stress; 12 and 13, oat under l00 mmol L^-1^ alkali stress.

**FIGURE 8:**
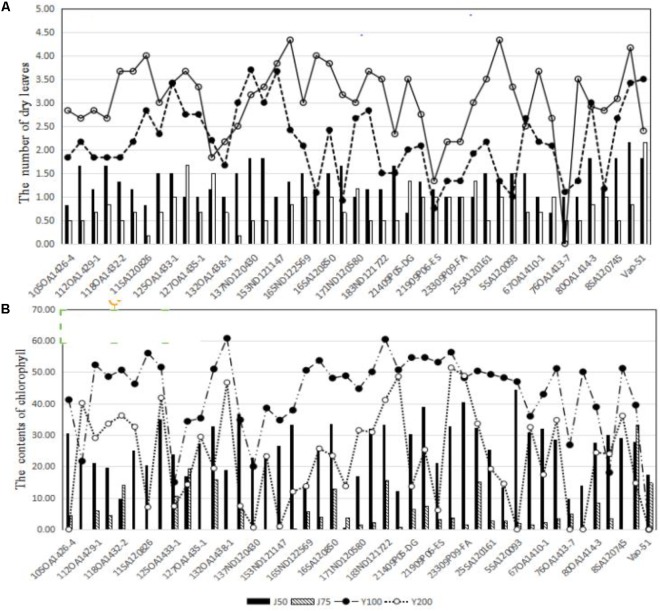
**(A)** The effects of salt and alkali stresses on the number of dry leaves for 43 oat genotypes. **(B)** The effects of salt and alkali stresses on chlorophyll contents for 43 oat genotypes. J50, 50 mmol L^-1^ alkali stress; J75, 75 mmol L^-1^ alkali stress; Y100, 100 mmol L^-1^ salt stress; Y200, 200 mmol L^-1^ salt stress.

**FIGURE 9:**
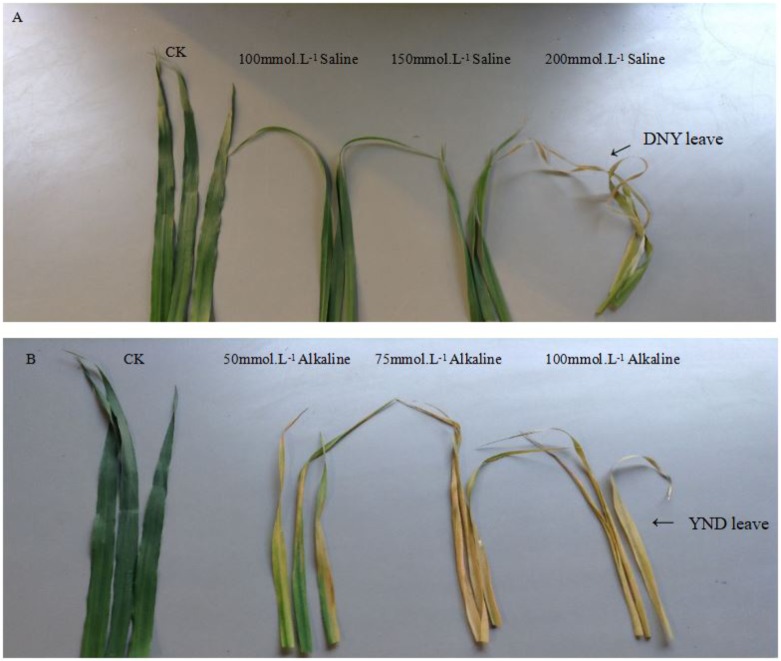
**(A)** The DNY leaves under salt stress. **(B)** The YND leaves under alkali stress. DNY leaves = dry but not yellow. YND leaves = yellow but not dry.

**FIGURE 10:**
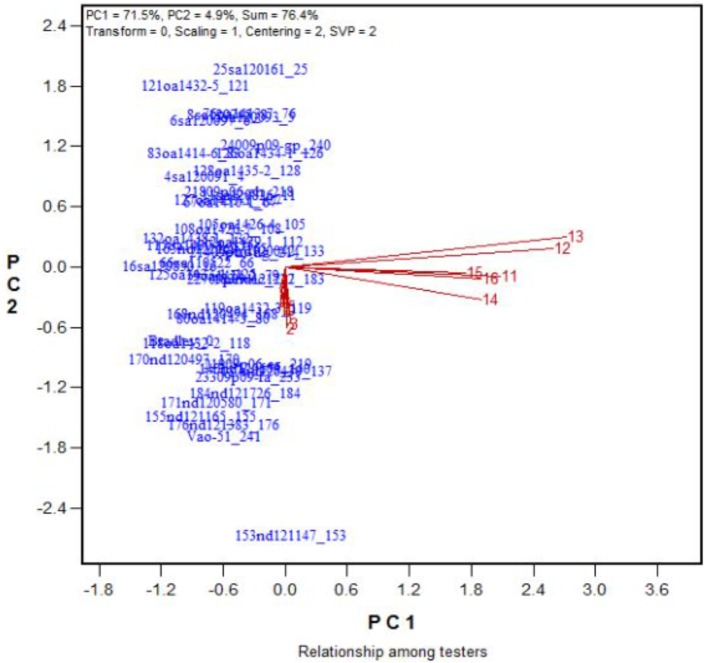
Effects of 16 treatments on the DNY leaves for 43 oat genotypes. See descriptions for each treatment in **Table 1**. DNY leaves = dry and not yellow.

**FIGURE 11:**
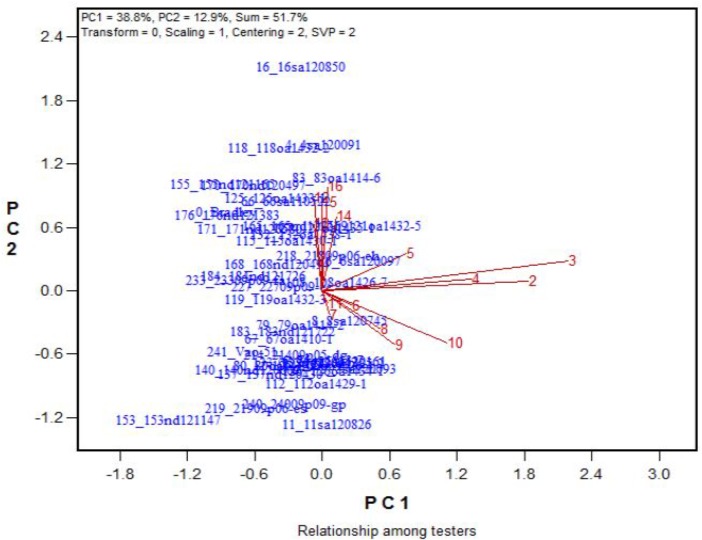
Effects of 16 treatments on the YND leaves for 43 oat genotypes. See descriptions for each treatment in **Table 1**. YND leaves = yellow and not dry.

Across the 43 genotypes tested in Experiment 3, chlorophyll content was lower under alkali stress than under salt stress. More yellow leaves were observed in the alkali treatments than in the salt treatments, while more dry leaves were observed in the salt treatments than in the alkali treatments (**Figures 6**–**8**), although the intensities of the salt treatments were more severe than the intensities of the alkali treatments. In addition, as shown in **Figure 9**, large numbers of leaves that were dry and not yellow (expressed as DNY) occurred in the salt treatments, whereas few of these leaves were found in the alkali treatments (**Table 7**). The cosine of the angle between the two treatments can be used to approximate the Pearson correlation between them. The DNY leaves responded differently to salinity and alkalinity, as indicated by the near right angles between the salinity levels (2, 3, 4, 5, 6, 7, 8, 9) and the alkalinity levels (11, 12, 13, 14, 15, 16). This result suggests that the effects of salinity on DNY leaves are different to those of alkalinity (**Figure 10**). Many leaves that were yellow and not dry (expressed as YND) appeared in the alkalinity treatments, whereas few of these leaves were found in the salinity treatments (**Figure 9** and **Table 7**). The YND leaves in the alkalinity treatment were not correlated with those in the salinity treatment, as indicated by the near right angles between the salinity levels (2, 3, 4, 5, 6, 7, 8, 9) and the alkalinity levels (12, 13, 14, 15, 16). This result suggests that the effects of salinity on YND leaves are different from the effects of alkalinity (**Figure 11**).

**Table 7:** The mean values of plant height, chlorophyll content, and numbers of tillers, panicles, DNY leaves, YND leaves, and grains per plant under different salinity and alkalinity levels.

Treatment ID	Number of kernels	Number of panicles	Height (cm)	Number of tillers	Chlorophyll	Number of DNY leaves	Number of YND leaves
Control	73.5	1.7	134.7	2.2	46.6	0.0	0.0
Saline levels		
S2	38.7	1.3	85.9	1.5	54.8	0.70	0.0
S3	37.2	1.3	77.2	1.4	53.3	0.85	0.0
S4	25.1	1.1	63.3	1.4	45.8	0.86	0.0
S5	24.8	1.0	56.8	0.7	53.4	0.88	0.0
S6	9.7	0.8	39.0	0.8	43.4	0.92	0.0
S7	7.0	0.7	32.5	0.7	36.5	0.93	0.0
S8	15.1	0.9	40.9	0.7	43.3	0.98	0.0
S9	5.7	0.6	27.0	0.0	22.4	0.99	0.0
S10	1.1	0.2	9.1	0.0	6.9	1.09	0.0
Alkaline levels		
A11	32.8	1.4	74.7	1.4	36.9	0.0	0.90
A12	10.4	1.0	53.2	0.0	25.5	0.0	0.97
A13	1.9	0.4	20.0	0.0	8.3	0.0	1.16
A14	0.6	0.5	24.7	0.3	7.8	0.0	1.12
A15	0.3	0.3	15.6	0.7	5.5	0.0	1.45
A16	0.4	0.2	11.3	0.5	3.2	0.0	1.47
A17	–	–	–	–	–	–	–
A18	–	–	–	–	–	–	–
A19	–	–	–	–	–	–	–
LSD (*P* = 0.05)	2.6	0.1	3.4	0.1	2.7	0.1	0.1


*The saline levels and alkaline levels are illustrated in **Table 1**. DNY leaves = dry and not yellow. YND leaves = yellow and not dry.*

All of the above results indicate that alkalinity mainly decreases chlorophyll content, while salinity mainly limits water absorption.

In experiment 3, the number of tillers and panicles, and the plant height were determined for each plant at the termination of the experiment. On average, the levels of all these traits decreased with increasing salinity, except for treatment S8 (**Table 7**). Some error was introduced in this treatment because the tray was slightly inclined and some plants in the tray were subjected to lower salinity levels than planned. The levels of these traits were all reduced by increased alkalinity more severely than by increased salinity. Data collection was not possible for the three most severe alkalinity levels (treatments 15–19; **Table 1**), as no plants survived to booting (**Table 7**).

### Physiological Index for Screening Salt and Alkali Tolerance

In Experiment 3, we compared the differences in chlorophyll content, panicle number, and plant height between tolerant and sensitive genotypes (**Tables 8**–**10**). As shown in **Tables 8**–**10**, the chlorophyll content of the tolerant genotypes (6SA120097, 16SA120850, 119OA1432-3, 121OA1432-5, 128OA1435-2, 112OA1429-1) were all higher than those of the sensitive genotypes (153ND121147, 137ND120430, 5SA120093, 83OA1414-6, 170ND120497, 67OA1410-1). This result indicates that chlorophyll content could be regarded as a physiological criterion for determining salt and alkali tolerance, and could be used to assist with the breeding of tolerant oat genotypes. The same phenomenon was not observed for panicle number and plant height.

**Table 8:** The effect of salt, alkali stresses on the chlorophyll content of tolerant and sensitive oat genotypes in experiment 3.

Variety	Mean	Mean/LSD5%	Class5%	Mean/LSD1%	Class1%
6SA120097	0.963	1.07	A	0.79	A
128OA1435-2	0.606	0.67	A	0.49	A
119OA1432-3	0.505	0.56	A	0.41	A
121OA1432-5	0.424	0.47	A	0.35	A
112OA1429-1	0.151	0.17	A	0.12	A
16SA120850	0.029	0.03		0.02	A
67OA1410-1	-0.015	-0.02		-0.01	A
83OA1414-6	-0.060	-0.07		-0.05	A
170ND120497	-0.435	-0.48		-0.36	
5SA120093	-0.514	-0.57		-0.42	
137ND120430	-0.784	-0.87		-0.64	
153ND121147	-0.868	-0.96		-0.71	


*Mean, the average value of chlorophyll content of each genotype under nine salinity and six alkalinity. If the difference in mean/LSD value between two genotypes is more than 1, then the difference in germination number between two genotypes is significant. Class5%, least significance at 5%; Class1%, least significance at 1%.*

**Table 9:** Effects of salt, alkali stresses on the panicle number of tolerant and sensitive oat genotypes in experiment 3.

Variety	Mean	Mean/LSD5%	Class5%	Mean/LSD1%	Class1%
6SA120097	1.37	2.02	A	1.49	A
67OA1410-1	0.746	1.1	A	0.81	A
16SA120850	0.419	0.62		0.45	
83OA1414-6	0.269	0.4		0.29	
121OA1432-5	0.160	0.24		0.17	
128OA1435-2	-0.068	-0.1		-0.07	
170ND120497	-0.210	-0.31		-0.23	
119OA1432-3	-0.353	-0.52		-0.38	
112OA1429-1	-0.408	-0.6		-0.44	
153ND121147	-0.636	-0.93		-0.69	
5SA120093	-0.636	-0.93		-0.69	
137ND120430	-0.659	-0.97		-0.71	


*Mean, the average value of panicle number each genotype under nine salinity and six alkalinity. If the difference in mean/LSD value between two genotypes is more than 1, then the difference in germination number between two genotypes is significant. Class5%, least significance at 5%; Class1%, least significance at 1%.*

**Table 10:** Effects of salt, alkali stresses on the plant height of tolerant and sensitive oat genotypes in experiment 3.

Variety	Mean	Mean/LSD5%	Class5%	Mean/LSD1%	Class1%
16SA120850	0.523	0.62		0.46	A
121OA1432-5	0.158	0.19		0.14	
128OA1435-2	0.133	0.16		0.12	
83OA1414-6	0.102	0.12		0.09	
67OA1410-1	0.058	0.07		0.05	
119OA1432-3	0.032	0.04		0.03	
112OA1429-1	-0.044	-0.05		-0.04	
137ND120430	-0.422	-0.5		-0.37	
170ND120497	-0.561	-0.67		-0.49	
5SA120093	-0.583	-0.69		-0.51	
153ND121147	-0.801	-0.95		-0.7	


*Mean, the average value of plant height each genotype under nine salinity and six alkalinity. If the difference in mean/LSD value between two genotypes is more than 1, then the difference in germination number between two genotypes is significant. Class5%, least significance at 5%; Class1%, least significance at 1%.*

## Discussion

### Saline and Alkaline Concentrations for Screening Oat Genotypes for Tolerance in Germination

Germination is a convenient test for salinity screening. Conflicting results have been reported regarding salinity tolerance during germination. Some researchers have found that there were relatively few differences among cultivars in terms of salt tolerance during germination (Malcolm et al., 2003). There seems to be little value in screening for salinity tolerance during germination (Munns and James, 2003). However, Oyiga et al. (2016) and Long et al. (2015) successfully screened for tolerant genotypes during the germination stage using various salinity concentrations, such as 100, 150, and 210 mmol L^-1^. This discrepancy may be attributed to differences in plant species and the salt concentrations used. Our results are consistent with the findings of Oyiga et al. (2016). In the present study, under the LC50 concentrations of salt (68.5 mmol L^-1^) and alkali (22.55 mmol L^-1^), significant differences in germination rates were detected among the 248 oat genotypes. Salt or alkali concentrations suitable for screening oat genotypes for tolerance must allow discrimination between genotypes. Using GGE-Biplot software, 248 genotypes were ranked based on their mean germination rates. The top 10 genotypes had significantly higher germination rates than the others. This result suggests that the LC50 concentrations of salt and alkali determined by the present study are effective for revealing genotypic variations in tolerance during germination.

### Correlation Between Tolerance During Germination and During Growth and Development

The criteria for salt tolerance is closely related to selection efficiency. In saline soil, oats with high yields could be regarded as the tolerant genotype. It is proposed that germination rate and the yield per plant are the two main factors responsible for inhibiting plant production under saline and alkaline soil conditions.

In addition, germination rate is a measure of tolerance under short-term salt stress, while yield per plant is a good representation of tolerance under long-term stress. Previous studies have mainly investigated short-term (a few hours or days) exposures of plants to salinity (Lee et al., 2005; Faiyue et al., 2012). However, long-term experiments are considered to be more reliable for screening crops for salinity tolerance because early responses to salt stress are mainly driven by the osmotic effect, while salt-specific effects require more time to develop (Zhu et al., 2016). Long-term exposure to salinity or alkalinity is more realistic and therefore more meaningful to plant breeding (Roy et al., 2014). The studies above led us to assume that tolerance to short-term salt stress may be unrelated to tolerance to long-term salt stress. To support this hypothesis, we used both germination rate and yield per plant as criteria for salt (or alkali) tolerance. This is one of the distinctive features of the current study. Our results demonstrated that tolerance during the germination stage and the growth stage were not correlated. Some genotypes that were salt-tolerant in the germination stage, such as 4SA120091, had low yields at the growth and development stage. We provide evidence that it is essential to examine both germination and growth stages to establish an effective screening method. This result is consistent with the results of Manaa et al. (2011), who claimed that the effects of short-term salinity on a plant are different from the effects of long-term salt stress.

### Salt and Alkali Treatments Suitable for Genotypic Screening for Tolerance During the Growth and Development Stage

One of the main reasons for the paucity of tolerant entries released is the lack of reliable and reproducible methods for identifying salt or alkali tolerance. In this study, we increased the screening efficiency in two ways, by: (1) improving the number of genotypes that are screened, and (2) improving the discriminating power of the salt (or alkali) treatments.

One feature of this study is the use of the cone-tainer system (**Figure 1**), which allowed for a large number of genotypes to be screened for salinity or alkalinity stress tolerance in a relatively small greenhouse space. The plastic “cone-tainer,” which is a new planting method, has been used to evaluate soybean genotypes for salt tolerance (Ledesma, 2016). We first used yield as the criterion for determining alkalinity and salinity tolerance using the plastic cone-tainer method. In a traditional pot experiment, for example, three replications of six oat genotypes would require 18 plastic pots occupying 2 m^2^. In this study, 15 oat genotypes with 6 replications could be fitted in one rack within 1.5 m^2^, and could be successfully evaluated for salt or alkali tolerance using the PC method. In addition, the PC planting method combines soil pots with hydroponics, and allowed 43 oat genotypes to be investigated in uniform salt or alkali solutions. Compared to pot experiments, the hydroponic system of the PC method created a uniform environment and allowed for the easy control of salinity and alkalinity levels. Taken together, these results show that the PC method is an easy, reliable, and highly efficient method for screening oat genotypes for salt tolerance.

Several studies have explored the salt intensity that is optimal for screening for tolerance (Matthew and Stacy, 2010; Peterson and Murphy, 2015). For example, of three NaCl concentrations tested (80, 120, and 160 mmol L^-1^) on four soybean genotypes, Ledesma et al. (2016) recommended 120 mmol L^-1^ as the best for demonstrating differences between tolerant and sensitive cultivars. However, these studies did not focus on the discriminating power of the salinity concentration, which is closely related to the screening efficiency. The optimal salt concentration for efficiently separating tolerant plants from sensitive ones among large numbers of cultivars has not yet been fully elucidated.

In scientific research, correct and effective data interpretation is as important as experimental design and implementation. In this study, we used GGE-Biplot software to determine the salt and alkali treatments with the best discriminating power. GGE-Biplot is a software program that used in crop variety trials (Yan et al., 2000, Yan and Kang, 2002; Yan, 2014). A biplot can be used to visualize three types of patterns: (1) relationships among the testers (treatments, traits, or their combination), (2) similarities or differences among genotypes, and (3) the discriminating power of the treatment or testing location. Crop breeders have used GGE to evaluate the performance of cultivars, and the discrimination and representativeness of locations (Zhang et al., 2016). For example, based on yield data from 13 durum wheat genotypes tested at four locations in northwestern Ethiopia, Abate et al. (2015) identified that the test location of Debretabor was the most discriminating environment for maximizing the variance among candidate cultivars compared to the locations of Adet and Simada. However, GGE-Biplot has not been used to screen oats for salt or alkali tolerance.

In this study, yield data from 43 oat genotypes tested at nine salinity and alkalinity levels were analyzed by GGE biplot. Treatment S6 (150 mmol L^-1^, 40 L) was the most discriminative salinity treatment, while A15 (75 mmol L^-1^, 40 L) was the most discriminative alkali treatment. To validate the established screening method, 262 genotypes were evaluated under these conditions. All of these genotypes were clearly differentiated in the S6 and A15 treatments In addition, GGE biplot can produce a numerical output of the differences among genotypes (in the form of a table) when a biplot is generated, and can automatically perform a variance analysis. For example, in experiment 4, the yields of genotypes 210, 199, 203, 56, and 103 were significantly higher than the yields of the other 257 genotypes. In total, 25 tolerant genotypes were found.

In the present work, we provide evidence that cone-tainers and GGE-biplot are effective tools for screening salt tolerant genotypes.

### Differences in the Effects of Salinity and Alkalinity on Oats

In northeastern China, nearly 70% of grasslands are alkalized, and these areas are expanding (Shi and Wang, 2005). It is now becoming clear that soil alkalization resulting from contamination by the alkaline salts NaHCO_3_ and Na_2_CO_3_ is a more severe problem than soil salinization caused by the neutral salts NaCl and Na_2_SO_4_. Alkali stress limits crop production more than neutral salt stress does (Wang et al., 2015). However, there are few studies on alkali stress (Campbell and Nishio, 2000; Hartung et al., 2002).

Several studies have reported that alkali stress inhibits plant growth (Yang et al., 2008), which is in agreement with our results. Guo et al. (2015) found that alkali stress has a stronger adverse effect on the distribution and accumulation of metabolites than salt stress. However, the reason why alkalinity stress inhibits growth and yield more than salt stress has not been elucidated. In this study, to solve this problem, differences in the mechanisms of responses to salt and alkali stress were compared. Alkalinity leads to more yellow leaves and lower chlorophyll content than salt stress. Large numbers of yellow leaves that were not dry appeared under alkali stress, but did not appear under salt stress. Many dry leaves that were not yellow occurred under salt stress, but did not occur under alkali stress. This phenomenon suggests that the adverse effects of salinity are mainly related to limitations in water absorption, whereas alkalinity primarily decreased chlorophyll content. Approximately 90–95% of crop biomass and yield are generated from photosynthesis (Wang et al., 2016), in which chlorophyll content plays a key role. Therefore, the greater adverse effects of alkalinity on yield were caused by damage to chlorophyll content, as compared to salinity.

Furthermore, our data revealed that the chlorophyll content in tolerant genotypes was higher than that in sensitive genotypes. This suggests that chlorophyll content could be considered the physiological criterion for both salt and alkali tolerance.

## Conclusion

This study made the following findings: (1) tolerance to salinity and alkalinity during germination and plant growth were not correlated, (2) the optimal saline and alkaline concentrations for selecting oat genotypes during germination were determined to be 68.5 and 22.5 mmol L^-1^, respectively, (3) saline and alkaline treatments suitable for selecting oat genotypes during the growth stage were identified as being S6 (150 mmol L^-1^ 40 L), A15 (75 mmol L^-1^ 40 L), respectively, (4) oat genotypes highly tolerant or sensitive to salinity and/or alkalinity were identified, (5) cone-tainer and GGE biplot software improved the efficiency of screening for salt and alkali tolerant genotypes, and (6) alkalinity mainly decreased chlorophyll content, while salinity mainly disrupted water absorption and balance. These results will be useful for further investigations into salt- and alkali-tolerance in crops.

## Author Contributions

JB and WY designed this experiments. JB conducted the experiment and wrote this paper. YW, QY, and JL participated in the experiment. CW helped take photos involved in this experiments. BM helped to revise this paper.

## Conflict of Interest Statement

The authors declare that the research was conducted in the absence of any commercial or financial relationships that could be construed as a potential conflict of interest.
