# Supplementary material for: Screening Oat Genotypes for Tolerance to Salinity and Alkalinity
Source: Front Plant Sci. 2018 Oct 2;9:1302. doi: 10.3389/fpls.2018.01302 (PMC6176118; doi:10.3389/fpls.2018.01302)
Supplement: Supplementary file 1 [file Table_1.DOC]

Table 1 The 248 varieties used in the experiment 2

| **ID** | **Variety** | **pedigree** | **ID** | **Variety** | **pedigree** |
| --- | --- | --- | --- | --- | --- |
| 3 | SA120088 | OT595/CDC Minstrel | 134 | ND120140 | 6/189_Dancer_CDC/ND071063_Drov_Jud_BG_HiF |
| 4 | SA120091 | OT595/CDC Minstrel | 135 | ND120142 | 6/189_Dancer_CDC/ND071063_Drov_Jud_BG_HiF |
| 5 | SA120093 | OT595/CDC Minstrel | 136 | ND120332 | 7/242_Diego-13/ND080726_Drov_MN00207 |
| 6 | SA120097 | OT595/CDC Minstrel | 137 | ND120430 | 19/67_Maida/ND051236 |
| 7 | SA120699 | Heinrich/CDC Seabiscuit | 138 | ND120485 | 22/132_MN05119/ND061975_(M2609) |
| 8 | SA120745 | SA080443/Atejo | 139 | ND120488 | 22/132_MN05119/ND061975_(M2609) |
| 9 | SA120756 | SA080443/Atejo | 140 | ND120609 | 31/4_ND000861/Beach |
| 10 | SA120794 | SA070972/14503 Cn | 141 | ND120624 | 31/26_ND000861/MN07210 |
| 11 | SA120826 | LAO-1104-075A1/OT3045 | 142 | ND120626 | 31/26_ND000861/MN07210 |
| 12 | SA120830 | LAO-1104-075A1/OT3045 | 143 | ND120628 | 31/26_ND000861/MN07210 |
| 13 | SA120831 | LAO-1104-075A1/OT3045 | 144 | ND120661 | 36/64_ND020965/ND051037 |
| 14 | SA120835 | LAO-1104-075A1/OT3045 | 145 | ND120662 | 37/55_ND020965/ND040250 |
| 15 | SA120841 | LAO-1104-075A1/OT3045 | 146 | ND120664 | 37/55_ND020965/ND040250 |
| 16 | SA120850 | LAO-1104-075A1/OT3045 | 147 | ND120712 | 39/199_ND021052/ND071313_Pc68 |
| 17 | SA120894 | SA070972/OT9001 | 148 | ND120716 | 41/52_ND021612/ND030374 |
| 20 | SA120993 | SA070972/SA051190 | 149 | ND120719 | 41/52_ND021612/ND030374 |
| 21 | SA121014 | SA070972/SA051190 | 150 | ND120996 | 49/81_ND030349/ND060183 |
| 22 | SA121094 | SA97404/CDC Morrison | 153 | ND121147 | 50/147_ND030364/ND070301 |
| 23 | SA120129 | OT590/CDC Dancer | 154 | ND121159 | 50/171_ND030364/ND070581 |
| 24 | SA120157 | OT590/CDC Dancer | 155 | ND121165 | 50/182_ND030364/ND070905 |
| 25 | SA120161 | OT590/CDC Dancer | 156 | ND121175 | 50/193_ND030364/ND071130_(Pc68) |
| 26 | SA120162 | OT590/CDC Dancer | 157 | ND121207 | 51/4_ND030365/Beach |
| 27 | SA120168 | OT590/CDC Dancer | 158 | ND121227 | 51/30_ND030365/Rockford |
| 28 | SA120175 | CDC Minstrel/OT594 | 159 | ND121233 | 51/37_ND030365/ND020965 |
| 29 | SA120181 | CDC Minstrel/OT594 | 160 | ND121249 | 51/99_ND030365/ND060507 |
| 30 | SA120192 | CDC Minstrel/OT594 | 161 | ND121408 | 61/6_ND050506/Dancer_CDC_Pc68 |
| 31 | SA121323 | Triactor/OT3040 | 162 | ND121416 | 61/37_ND050506/ND020965_Pc68 |
| 32 | SA121340 | Triactor/OT3040 | 163 | ND121444 | 70/17_ND051312/Leggett |
| 33 | SA121348 | Triactor/OT3040 | 164 | ND121730 | 108/17_ND060897/Leggett |
| 34 | SA121352 | Triactor/OT3040 | 165 | ND122569 | 256/49_SA060422/ND030349 |

**Continue Table 1**

| **ID** | **Variety** | **pedigree** | **ID** | **Variety** | **pedigree** |
| --- | --- | --- | --- | --- | --- |
| 37 | SA120236 | OT7053/CDC ProFi | 166 | ND120043 | 6/52_Dancer_CDC/ND030374 |
| 38 | SA120240 | OT7053/CDC ProFi | 167 | ND120093 | 6/144_Dancer_CDC/ND070212_021612_RD95031 |
| 39 | SA121474 | OT3031/OT3051 | 168 | ND120494 | 24/70_MN07104/ND051312 |
| 40 | SA121482 | OT3031/OT3051 | 169 | ND120496 | 24/70_MN07104/ND051312 |
| 41 | SA121501 | OT3031/OT3051 | 170 | ND120497 | 24/70_MN07104/ND051312 |
| 42 | SA121962 | OT2053/OT3045 | 171 | ND120580 | 30/140_Rockford/ND070183 |
| 43 | SA121034 | OT7053/SA051190 | 172 | ND121119 | 50/22_ND030364/MN05119 |
| 44 | SA121044 | OT7053/SA051190 | 173 | ND121120 | 50/22_ND030364/MN05119 |
| 45 | SA121046 | OT7053/SA051190 | 174 | ND121184 | 50/210_ND030364/ND071482 |
| 46 | SA121810 | OT3033/OT3028 | 175 | ND121278 | 51/201_ND030365/ND071332 |
| 47 | SA121812 | OT3033/OT3028 | 176 | ND121383 | 52/139_ND030374/ND070182 |
| 48 | SA121815 | OT3033/OT3028 | 177 | ND121428 | 70/9_ND051312/Furlong_AC |
| 49 | SA121820 | OT3033/OT3028 | 178 | ND121432 | 70/9_ND051312/Furlong_AC |
| 50 | SA120318 | OT3031/OT2055 | 179 | ND121435 | 70/9_ND051312/Furlong_AC |
| 51 | SA120320 | OT3031/OT2055 | 180 | ND121437 | 70/17_ND051312/Leggett |
| 53 | SA120355 | OT3041/OT2055 | 181 | ND121538 | 80/36_ND060182_(Danc)/ND020965 |
| 54 | SA120259 | OT3031/LAO-1042-044 | 182 | ND121613 | 89/38_ND060342/ND020971 |
| 55 | SA120263 | OT3031/LAO-1042-044 | 183 | ND121722 | 108/17_ND060897/Leggett |
| 56 | SA120274 | OT3031/LAO-1042-044 | 184 | ND121726 | 108/17_ND060897/Leggett |
| 57 | SA121535 | CDC Big Brown/OT595 | 185 | ND121805 | 122/41_ND061519/ND021612 |
| 58 | SA121541 | CDC Big Brown/OT595 | 186 | ND121817 | 127/6_ND061673/Dancer_CDC |
| 59 | SA122023 | SA060422/CDC Big Brown | 187 | ND122047 | 139/248_ND070182/ND970651 |
| 60 | SA121620 | OT595/OT3031 | 188 | ND122066 | 144/41_ND070212/ND021612_RD |
| 61 | SA121628 | OT595/OT3031 | 189 | ND122218 | 171/9_ND070581/Furlong_AC |
| 62 | SA121700 | OT3033/Bradley | 190 | ND122302 | 200/39_ND071330/ND021052 |
| 63 | SA121712 | OT3033/Bradley | 191 | ND122380 | 216/9_ND071570/Furlong_AC |
| 64 | SA121758 | OT2055/CDC Seabiscuit | 192 | ND122394 | 216/171_ND071570/ND070581 |
| 65 | SA121775 | OT2055/CDC Seabiscuit | 194 | ND122542 | 251/68_ND991293/ND051306 |
| 66 | SA110522 | Stainless/CDC Minstrel | 195 | ND122566 | 256/49_SA060422/ND030349 |
| 67 | OA1410-1 | Navarro/ VAO44 | 196 | ND122568 | 256/49_SA060422/ND030349 |
| 68 | OA1411-1 | OA1196-3/OA1251-1 | 197 | ND122585 | 260/49_SA060832/ND030349 |
| 69 | OA1412-1 | OA1272-1/SA050505 | 198 | ND122586 | 260/49_SA060832/ND030349 |
| 71 | OA1413-2 | OA1228-1/SA050505//04P07B-GT3D/HiFi | 199 | 09P10-KJ1 | OT7053/CDC Morrison |

| **ID** | **Variety** | **pedigree** | **ID** | **Variety** | **pedigree** |
| --- | --- | --- | --- | --- | --- |
| 72 | OA1413-3 | OA1228-1/SA050505//04P07B-GT3D/HiFi | 200 | 09P10-PK4 | OT7053/CDC Morrison |
| 73 | OA1413-4 | OA1228-1/SA050505//04P07B-GT3D/HiFi | 201 | 09P01-EP | OT590/OT2055 |
| 74 | OA1413-5 | OA1228-1/SA050505//04P07B-GT3D/HiFi | 202 | 09P02-BZ | Stainless/01G04-CC3E |
| 75 | OA1413-6 | OA1228-1/SA050505//04P07B-GT3D/HiFi | 203 | 09P02-CB | Stainless/01G04-CC3E |
| 76 | OA1413-7 | OA1228-1/SA050505//04P07B-GT3D/HiFi | 204 | 09P02-CC | Stainless/01G04-CC3E |
| 77 | OA1413-8 | OA1228-1/SA050505//04P07B-GT3D/HiFi | 205 | 09P02-CS | Stainless/01G04-CC3E |
| 78 | OA1414-1 | HiFi/OA1189-4//OA1228-1/04P07B-GT3D | 206 | 09P08-DB | Stainless/01G04-CC3E |
| 79 | OA1414-2 | HiFi/OA1189-4//OA1228-1/04P07B-GT3D | 207 | 09P02-FG | Stainless/01G04-CC3E |
| 80 | OA1414-3 | HiFi/OA1189-4//OA1228-1/04P07B-GT3D | 208 | 09P02-FX | Stainless/01G04-CC3E |
| 81 | OA1414-4 | HiFi/OA1189-4//OA1228-1/04P07B-GT3D | 209 | 09P02-HB | Stainless/01G04-CC3E |
| 82 | OA1414-5 | HiFi/OA1189-4//OA1228-1/04P07B-GT3D | 210 | 09P02-HH | Stainless/01G04-CC3E |
| 83 | OA1414-6 | HiFi/OA1189-4//OA1228-1/04P07B-GT3D | 211 | 09P02-HM | Stainless/01G04-CC3E |
| 84 | OA1415-1 | OA1250-1/MN07205//rigodon/HiFi | 212 | 09P02-HR | Stainless/01G04-CC3E |
| 85 | OA1415-2 | OA1250-1/MN07205//rigodon/HiFi | 213 | 09P03-JD | 05P19-AS4E//05P19-AH1C/Pc101 |
| 86 | OA1415-3 | OA1250-1/MN07205//rigodon/HiFi | 214 | 09P05-DG | Stainless*2/Pc101 |
| 87 | OA1415-4 | OA1250-1/MN07205//rigodon/HiFi | 215 | 09P06-AU | OT9001/OT2061 |
| 88 | OA1416-1 | OA1250-1/MN07205//OA1228-1/SA050505 | 216 | 09P06-BA | OT9001/OT2061 |
| 89 | OA1417-1 | OA1251-1/FL03167BSB-71-B-s1//OA1228-1/SA050505 | 217 | 09P06-BB | OT9001/OT2061 |
| 90 | OA1418-1 | OA1251-1/FL03167BSB-71-B-s1//04P07B-GT3D/HiFi | 218 | 09P06-EH | OT9001/OT2061 |
| 91 | OA1418-2 | OA1251-1/FL03167BSB-71-B-s1//04P07B-GT3D/HiFi | 219 | 09P06-ES | OT9001/OT2061 |

**Continue Table 1**

**Continue Table 1**

| **ID** | **Variety** | **pedigree** | **ID** | **Variety** | **pedigree** |
| --- | --- | --- | --- | --- | --- |
| 92 | OA1419-1 | HiFi/OA1189-4//Navaro/OA1202-1 | 220 | 09P07-DT | OT9001/OT7055 |
| 93 | OA1419-2 | HiFi/OA1189-4//Navaro/OA1202-1 | 221 | 09P08-AP | OT596/OT7055 |
| 94 | OA1419-3 | HiFi/OA1189-4//Navaro/OA1202-1 | 222 | 09P09-AA | OT596/OT2061 |
| 95 | OA1420-1 | HiFi/OA1256-1//MN07205/OA1228-1 | 223 | 09P09-BE | OT596/OT2061 |
| 96 | OA1420-2 | HiFi/OA1256-1//MN07205/OA1228-1 | 224 | 09P09-BP | OT596/OT2061 |
| 97 | OA1421-1 | HylCm404/04P07B-GT3D//OA1251-1/HiFi | 225 | 09P09-BV | OT596/OT2061 |
| 98 | OA1422-1 | HiFi/OA1256-1//SA04213 | 226 | 09P09-EA | OT596/OT2061 |
| 99 | OA1423-1 | OA1228-1/04P07B-GT3D//ND30365 | 227 | 09P09-EC | OT596/OT2061 |
| 100 | OA1424-1 | unknown//unknown | 228 | 09P09-EE | OT596/OT2061 |
| 101 | OA1425-1 | HiFi1/OA1225-2 | 229 | 09P09-EF | OT596/OT2061 |
| 102 | OA1426-1 | MN08261/OA1306-1 | 230 | 09P09-EN | OT596/OT2061 |
| 103 | OA1426-2 | MN08261/OA1306-1 | 231 | 09P09-EU | OT596/OT2061 |
| 104 | OA1426-3 | MN08261/OA1306-1 | 232 | 09P09-EX | OT596/OT2061 |
| 105 | OA1426-4 | MN08261/OA1306-1 | 233 | 09P09-FA | OT596/OT2061 |
| 106 | OA1426-5 | MN08261/OA1306-1 | 234 | 09P09-FE | OT596/OT2061 |
| 107 | OA1426-6 | MN08261/OA1306-1 | 235 | 09P09-FH | OT596/OT2061 |
| 108 | OA1426-7 | MN08261/OA1306-1 | 239 | 09P09-GA | OT596/OT2061 |
| 109 | OA1427-1 | OA1225-2/OA1256-1 | 240 | 09P09-GP | OT596/OT2061 |
| 110 | OA1427-2 | OA1225-2/OA1256-1 | 241 | 05P17-OA35A | B548.4.2 |
| 111 | OA1428-1 | OA1228-1/HiFi2 | 242 | OSP17-OA35-ES29 | OT7070 INH Breeder 2012 #29 |
| 112 | OA1429-1 | OA1231-2-1/OA1256-1 | 244 | Dancer | - |
| 113 | OA1429-2 | OA1231-2-1/OA1256-1 | 245 | leggett | - |
| 114 | OA1429-3 | OA1231-2-1/OA1256-1 | 246 | morgan | - |
| 115 | OA1430-1 | OA1285-1/OA1231-2-1 | 247 | orrin | - |
| 116 | OA1431-1 | OA1285-1/SA04213 | 248 | souris | - |

**Continue Table 1**

| **ID** | **Variety** | **pedigree** | **ID** | **Variety** | **pedigree** |
| --- | --- | --- | --- | --- | --- |
| 117 | OA1432-1 | OA1285-1/SA04213 | 123 | OA1432-7 | OA1285-1/SA04213 |
| 118 | OA1432-2 | OA1285-1/SA04213 | 124 | OA1432-8 | OA1285-1/Bradley |
| 119 | OA1432-3 | OA1285-1/SA04213 | 125 | OA1433-1 | OA1285-1/SA050174 |
| 121 | OA1432-5 | OA1285-1/SA04213 | 126 | OA1434-1 | OA1285-1/SA050174 |
| 122 | OA1432-6 | OA1285-1/SA04213 | 127 | OA1435-1 | OA1285-1/OA1225-2 |
| 128 | OA1435-2 | OA1285-1/OA1225-2 | 131 | OA1437-2 | SA04213/MN06203 |
| 129 | OA1436-1 | OA1306-1/Bradley | 132 | OA1438-1 | SA04213/OA1306-1 |
| 130 | OA1437-1 | SA04213/MN06203 | 133 | ND120042 | 6/50_Dancer_CDC/ND030364 |
