# Supplementary material for: Screening Oat Genotypes for Tolerance to Salinity and Alkalinity
Source: Front Plant Sci. 2018 Oct 2;9:1302. doi: 10.3389/fpls.2018.01302 (PMC6176118; doi:10.3389/fpls.2018.01302)
Supplement: Supplementary file 2 [file Table_2.DOC]

**Table 2** The 262 varieties used in the experiment 4

| **ID** | **Variety** | **pedigree** | **ID** | **Variety** | **pedigree** |
| --- | --- | --- | --- | --- | --- |
| 1 | OA1439-1 | HiFi/OA1256-1//SA04213 | 132 | SA130728 | CDC Morrison/CDC Big Brown |
| 2 | OA1440-1 | MN06213/OA1253-1 | 133 | SA130733 | CDC Morrison/CDC Big Brown |
| 3 | OA1440-2 | MN06213/OA1253-1 | 134 | SA130772 | CDC Seabiscuit/06 T-503-07-022 |
| 4 | OA1441-1 | OA1306-1/MN06203 | 135 | SA130794 | OT3063/CDC Morrison |
| 5 | OA1441-2 | OA1306-1/MN06203 | 136 | SA130811 | OT3063/CDC Morrison |
| 6 | OA1441-3 | OA1306-1/MN06203 | 137 | SA130882 | OT3057/SA090139 |
| 7 | OA1441-4 | OA1306-1/MN06203 | 138 | SA130898 | OT3057/SA090139 |
| 8 | OA1442-1 | SA04213/OA1306-1 | 139 | SA130934 | SW Betania/OT3057 |
| 9 | OA1442-2 | SA04213/OA1306-1 | 140 | SA130937 | SW Betania/OT3057 |
| 10 | OA1443-1 | SA060123/Dieter | 141 | SA131429 | OT3045/SA060198 |
| 11 | OA1444-1 | SA04213/OA1271-3 | 142 | SA131441 | OT3045/SA060198 |
| 12 | OA1444-2 | SA04213/OA1271-3 | 143 | SA130493 | SA97404/CDC Morrison |
| 13 | OA1444-3 | SA04213/OA1271-3 | 144 | SA130502 | SA97404/CDC Morrison |
| 14 | OA1444-4 | SA04213/OA1271-3 | 145 | SA130512 | SA97404/CDC Morrison |
| 15 | OA1444-5 | SA04213/OA1271-3 | 146 | SA130575 | Mn07205/CDC Seabiscuit |
| 16 | OA1444-6 | SA04213/OA1271-3 | 147 | SA130964 | OT3053/OT7062 |
| 17 | OA1445-1 | TX07CS1948/OA1317-1 | 148 | SA130972 | OT3053/OT7062 |
| 18 | OA1446-1 | Profi/OA1251-1-5 | 149 | SA131005 | OT3063/Triactor |
| 19 | OA1447-1 | 09350-61-1/OA1285-1-12 | 150 | SA131009 | OT3063/Triactor |
| 20 | OA1448-1 | Dieter/OA1251-1-5 | 151 | SA131010 | OT3063/Triactor |
| 21 | OA1448-2 | Dieter/OA1251-1-5 | 152 | SA131018 | OT3063/Triactor |
| 22 | OA1449-1 | SA04213/OA1301-1 | 153 | SA131048 | OT3063/Triactor |
| 23 | OA1450-1 | X9414-1/SA060123 | 154 | SA131689 | CDC Nasser/Big Brown |
| 24 | OA1451-1 | X9503-1/OA1301-1 | 155 | 10P05A-169E2 | OT7053/CDC Morrison//AC Morgan/Stainless |
| 25 | OA1452-1 | TX07CS1948/Marion | 156 | 10P05A-176D1 | OT7053/CDC Morrison//AC Morgan/Stainless |
| 26 | OA1453-1 | SA060123/04P07B-GN1C | 157 | 10P05A-176D2 | OT7053/CDC Morrison//AC Morgan/Stainless |
| 27 | OA1454-1 | SA04213/04P07B-FR3B | 158 | 10P05A-176D3 | OT7053/CDC Morrison//AC Morgan/Stainless |
| 28 | OA1455-1 | SA060123/09453-0-22 | 159 | 10P10A-032E5 | Stride/CDC Morrison |
| 29 | OA1456-1 | 060ANS28/SA060123 | 160 | 10P10C-008A5 | Stride/CDC Morrison |
| 30 | OA1456-2 | 060ANS28/SA060123 | 161 | 10P10C-043E3 | Stride/CDC Morrison |
| 31 | OA1457-1 | SA070712/OA1317-1 | 162 | 10P10D-030E4 | Stride/CDC Morrison |
| 32 | 10W60-382 | SA04213/TX07CS-1948 | 163 | 10P10D-047D3 | Stride/CDC Morrison |
| 33 | 10W60-103 | SA04213/TX07CS-1948 | 164 | 10P10D-059B4 | Stride/CDC Morrison |
| 34 | 10W60-241 | SA04213/TX07CS-1948 | 165 | 10P10D-059B5 | Stride/CDC Morrison |

**Continue Table 2**

| **ID** | **Variety** | **pedigree** | **ID** | **Variety** | **pedigree** |
| --- | --- | --- | --- | --- | --- |
| 35 | OA1331-5-0-21 | OA1331-5 selection | 166 | 10P02B-086 | OT9001/OT7055//AC Morgan/Stainless |
| 36 | 11W08-2 | OA1251-1A/OA1250-1 | 167 | 10P03A-005 | OT596/OT7055//AC Morgan/Stainless |
| 37 | 11W08-10 | OA1251-1A/OA1250-1 | 168 | 10P03A-024 | OT596/OT7055//AC Morgan/Stainless |
| 38 | 11W08-17 | OA1251-1A/OA1250-1 | 169 | 10P03A-044 | OT596/OT7055//AC Morgan/Stainless |
| 39 | 11W10-4 | Morrison/OA1301-1W-6 | 170 | 10P01A-005 | OT9001/OT2061//AC Morgan/Stainless |
| 40 | 11W09-2 | Morrison/OA1250-1 | 171 | 10P01A-077 | OT9001/OT2061//AC Morgan/Stainless |
| 41 | 11W16-8 | OA1251-1A/Orrin | 172 | 10P04A-012 | OT596/OT2061//AC Morgan/Stainless |
| 42 | 11W11-6 | Morrison/Orrin | 173 | 10P04A-014 | OT596/OT2061//AC Morgan/Stainless |
| 43 | 11W14-13 | Morrison/SA060123 | 174 | 10P04A-084 | OT596/OT2061//AC Morgan/Stainless |
| 44 | 11W51-20 | 06ANS28/OA1251-1A | 175 | 10P04A-085 | OT596/OT2061//AC Morgan/Stainless |
| 45 | 11W17-2 | OA1251-1A/OA1331-6 | 176 | 10P08B-012 | CDC Nasser/OT7065 |
| 46 | 11W55-25 | OA1285-1-12/OA1251-1A | 177 | 10P12A-087 | OT2072/Stride |
| 47 | 11W10-6 | Morrison/OA1301-1W-6 | 178 | 10P12B-006 | OT2072/Stride |
| 48 | 11W55-26 | OA1285-1-12/OA1251-1A | 179 | 10P12B-008 | OT2072/Stride |
| 49 | 11W13-4 | Morrison/Florida167 | 180 | 10P12B-035 | OT2072/Stride |
| 50 | 11W11-5 | Morrison/Orrin | 181 | 10P12B-045 | OT2072/Stride |
| 51 | 11W55-17 | OA1285-1-12/OA1251-1A | 182 | 10P12C-012 | OT2072/Stride |
| 52 | 11W19-29 | Morrison/OA1331-6 | 183 | 10P12C-022 | OT2072/Stride |
| 53 | 11W55-16 | OA1285-1-12/OA1251-1A | 184 | 10P12C-043 | OT2072/Stride |
| 54 | 11W10-3 | Morrison/OA1301-1W-6 | 185 | 10P07A-051 | AC Morgan/Stainless//AC Morgan*2/CIav2014:BC1F1 |
| 55 | 11W32-5 | OA1347-1/OA1341-1-2 | 186 | 10P07A-083 | AC Morgan/Stainless//AC Morgan*2/CIav2014:BC1F1 |
| 56 | 11W13-1 | Morrison/Florida167 | 187 | 07P15A-028 | SW Betania/Stainless |
| 57 | 11W14-16 | Morrison/SA060123 | 188 | 07P15A-032 | SW Betania/Stainless |
| 58 | 11W11-9 | Morrison/Orrin | 189 | 07P15B-026 | SW Betania/Stainless |
| 59 | 11W56-7 | Morrison/OA1347-3 | 190 | 07P15B-029 | SW Betania/Stainless |
| 60 | 11W12-4 | OA1250-1/FL03167-BSBS-3 | 191 | 10P06A-018 | AC Morgan/HiFi Reselected |
| 61 | 11W10-2 | Morrison/OA1301-1W-6 | 192 | 10P06A-036 | AC Morgan/HiFi Reselected |
| 62 | 11W13-10 | Morrison/Florida167 | 193 | 10P06A-076 | AC Morgan/HiFi Reselected |

**Continue Table 2**

| **ID** | **Variety** | **pedigree** | **ID** | **Variety** | **pedigree** |
| --- | --- | --- | --- | --- | --- |
| 63 | 11W44-5 | OA1341-1-4/OA1331-6 | 194 | 10P06A-082 | AC Morgan/HiFi Reselected |
| 64 | 11W44-8 | OA1341-1-4/OA1331-6 | 195 | 10P06A-085 | AC Morgan/HiFi Reselected |
| 65 | 11W44-9 | OA1341-1-4/OA1331-6 | 196 | 10P06A-142 | AC Morgan/HiFi Reselected |
| 66 | 11W56-5 | Morrison/OA1347-3 | 197 | 10P06A-225 | AC Morgan/HiFi Reselected |
| 67 | 11W03-17 | OA1250-1/09350-189 | 198 | 12QB03-BF | Florida F4s (12QION Entries 163-217) |
| 68 | 11W53-49 | OA1251-1A/OA1357-2 | 199 | ND130775 | 66/3 ND060182/04P07B-GN1C |
| 69 | 11W18-16 | OA1251-1A/OA1260-1II | 200 | ND131519 | 185/9 ND081029/CRRSRR3 |
| 70 | 11W18-18 | OA1251-1A/OA1260-1II | 201 | ND130020 | 9/61 CRRSRR3/ND051306 |
| 71 | 11W18-22 | OA1251-1A/OA1260-1II | 202 | ND130662 | 60/26 ND051236/MN07210 |
| 72 | 11W31-9 | Morrison/OA1357-2 | 203 | ND130776 | 66/3 ND060182/04P07B-GN1C |
| 73 | 11W37-2 | Morrison/OA1306-1 | 204 | ND130793 | 67/2 ND060183/04P07A-BS5C |
| 74 | 11W37-7 | Morrison/OA1306-1 | 205 | ND132448 | 216/171 ND071570/ND070581 |
| 75 | 11W43-6 | OA1341-1-4/OA1331-5 | 206 | ND130182 | 19/10 Leggett/ND091846 |
| 76 | 11W44-1 | OA1341-1-4/OA1331-6 | 207 | ND130452 | 44/200 ND030349/ND081674 |
| 77 | 11W45-11 | OA1251-1A/OA1331-5 | 208 | ND131510 | 182/19 ND080931/Leggett |
| 78 | 11W50-1 | SA04213/TX07CS2140 | 209 | ND131874 | 7/199 Diego-13/ND071313 a N Pc-91 Pc-91Maid |
| 79 | 11W51-7 | 06ANS28/OA1251-1A | 210 | ND131936 | 24/70 MN07104/ND051312 |
| 80 | 11W51-15 | 06ANS28/OA1251-1A | 211 | ND132123 | 50/182 ND030364/ND070905 Maida 232 |
| 81 | 11W53-16 | OA1251-1A/OA1357-2 | 212 | ND132527 | 260/49 SA060832/ND030349 |
| 82 | 11W53-25 | OA1251-1A/OA1357-2 | 213 | ND130110 | 11/89 Dancer CDC/ND070497 |
| 83 | 11W53-26 | OA1251-1A/OA1357-2 | 214 | ND130268 | 26/53 MN07210/ND050427 |
| 84 | 11W53-28 | OA1251-1A/OA1357-2 | 215 | ND130269 | 26/53 MN07210/ND050427 |
| 85 | 11W55-5 | OA1285-1-12/OA1251-1A | 216 | ND130327 | 32/84 ND000824/ND070395 (Morgan) |
| 86 | 11W55-6 | OA1285-1-12/OA1251-1A | 217 | ND130601 | 54/138 ND050490/ND080117 |
| 87 | 11W55-14 | OA1285-1-12/OA1251-1A | 218 | ND130664 | 60/26 ND051236/MN07210 |
| 88 | 11W57-15 | OA1357-2/SA070712 | 219 | ND130788 | 67/2 ND060183/04P07A-BS5C |
| 89 | 11W57-23 | OA1357-2/SA070712 | 220 | ND131223 | 145/19 ND080217/Leggett |
| 90 | 11W59-3 | SA070712/OA1251-1A | 221 | ND131475 | 173/27 ND080609/Monida |
| 91 | 11W64-27 | OA1347-3/OA1357-2 | 222 | ND131479 | 175/66 ND080726/ND060182 |
| 92 | 11W66-2 | OA1225-2/OA1347-3 | 223 | ND131507 | 182/19 ND080931/Leggett |
| 93 | 11W79-1 | Trophy/Orrin | 224 | ND131514 | 183/35 ND080946/ND020290 (PO 808) |

**Continue Table 2**

| **ID** | **Variety** | **pedigree** | **ID** | **Variety** | **pedigree** |
| --- | --- | --- | --- | --- | --- |
| 94 | 10W44-1-13 | SA060123/04P07B-GN1C | 225 | ND131634 | 196/8 ND081475/CRRSRR2 |
| 95 | SA130006 | Heinrich/CDC Seabiscuit | 226 | ND131653 | 204/17 ND081727/Jerry |
| 96 | SA130027 | SA080443/Atejo | 227 | ND131749 | 219/107 Pinnacle AC/ND071313 |
| 97 | SA130688 | Heinrich/OT3050 | 228 | ND132420 | 186/9 ND071017/Furlong AC |
| 98 | SA131101 | SW080802/OT3045 | 229 | ND130035 | 10/15 ND091846/HiFi |
| 99 | SA131108 | SW080802/OT3045 | 230 | ND130077 | 10/111 ND091846/ND071570 |
| 100 | SA131113 | SW080802/OT3045 | 231 | ND130093 | 11/62 Dancer CDC/ND051312 |
| 101 | SA131135 | SW081506/CDC Seabiscuit | 232 | ND130111 | 11/89 Dancer CDC/ND070497 |
| 102 | SA131137 | SW081506/CDC Seabiscuit | 233 | ND130143 | 14/71 Furlong AC/ND060342 |
| 103 | SA131566 | CDC Seabiscuit/CDC Big Brown | 234 | ND130340 | 32/103 ND000824/ND071128 |
| 104 | SA131575 | CDC Seabiscuit/CDC Big Brown | 235 | ND130357 | 35/93 ND020290 (PO 808)/ND070581 |
| 105 | SA131615 | CDC Big Brown/SA060605 | 236 | ND130492 | 45/210 ND030365/ND081834 |
| 106 | SA131618 | CDC Big Brown/SA060605 | 237 | ND130579 | 53/54 ND050427/ND050490 |
| 107 | SA131763 | CDC Ruffian/99AB11787 | 238 | ND130607 | 57/111 ND051037/ND071570 |
| 108 | SA131839 | CDC Ruffian/Atejo | 239 | ND130784 | 66/3 ND060182/04P07B-GN1C |
| 109 | SA131873 | CDC Ruffian/Atejo | 240 | ND130864 | 71/25 ND060342/MN07204 |
| 110 | SA131880 | Pajaz/CDC Ruffian | 241 | ND130880 | 75/15 ND061097/HiFi |
| 111 | SA131896 | Pajaz/CDC Ruffian | 242 | ND130884 | 75/15 ND061097/HiFi |
| 112 | SA131897 | Pajaz/CDC Ruffian | 243 | ND130888 | 75/15 ND061097/HiFi |
| 113 | SA131905 | Pajaz/CDC Ruffian | 244 | ND131474 | 173/27 ND080609/Monida |
| 114 | SA131923 | SA081656/Viviana | 245 | ND131488 | 175/83 ND080726/ND070388 |
| 115 | SA131930 | SA081656/Viviana | 246 | ND131504 | 182/19 ND080931/Leggett |
| 116 | SA131955 | OT9001/OA1196-3 | 247 | ND131700 | 215/79 ND082051/ND061975 (Otana//M2609/Otana) |
| 117 | SA131995 | OT2069/SA070860 | 248 | ND131713 | 216/14 ND082052/Furlong AC |
| 118 | SA132015 | OT2069/SA070860 | 249 | ND131745 | 219/107 Pinnacle AC/ND071313 |
| 119 | SA132045 | OT9001/OT3060 | 250 | ND132180 | 70/17 ND051312/Leggett |
| 120 | SA130397 | OT7053/SA051190 | 251 | ND132181 | 70/17 ND051312/Leggett |
| 121 | SA130460 | SA080443/OT7053 | 252 | ND132184 | 70/17 ND051312/Leggett |
| 122 | SA130468 | SA080443/OT7053 | 253 | ND132186 | 70/17 ND051312/Leggett |
| 123 | SA130112 | Bajka/SA070972 | 254 | ND132274 | 120/17 ND061427/Leggett |
| 124 | SA130186 | LAO-1104-075A1/OT3045 | 255 | ND132392 | 164/81 ND070500/ND060183 |
| 125 | SA130278 | IA2130-2-2/SA071225 | 256 | ND132408 | 171/9 ND070581/Furlong AC |
| 126 | SA130308 | OT9001/SA071225 | 257 | ND132441 | 213/45 ND071521/ND030288 |
| 127 | SA131277 | 14340 Cn3/1/CDC Big Brown | 258 | ND132523 | 258/51 SA060544/ND030365 |
| 128 | SA131379 | OT9001/CDC Big Brown | 259 | ND132526 | 260/49 SA060832/ND030349 |

**Continue Table 2**

| **ID** | **Variety** | **pedigree** | **ID** | **Variety** | **pedigree** |
| --- | --- | --- | --- | --- | --- |
| 129 | SA130372 | SA070972/SA051190 | 260 | ND132528 | 260/49 SA060832/ND030349 |
| 130 | SA130378 | SA070972/SA051190 | 261 | ND132603 | 09262 120/17 ND061427/Leggett |
| 131 | SA130718 | CDC Morrison/CDC Big Brown | 262 | ND132615 | 09311 171/9 ND070581/Furlong AC |
