# Supplementary material for: Screening Oat Genotypes for Tolerance to Salinity and Alkalinity
Source: Front Plant Sci. 2018 Oct 2;9:1302. doi: 10.3389/fpls.2018.01302 (PMC6176118; doi:10.3389/fpls.2018.01302)
Supplement: Supplementary file 3 [file Table_3.DOC]

**Table 3** Germination rates and grain number of 43 genotypes under salinity and alkalinity stress

|  |  | Germination rate (%) under | | Number of grains under | |
| --- | --- | --- | --- | --- | --- |
| Entry ID | Name | 22.5mmol.L^-1^ salt | 68.5mmol.L^-1^ alkali | Salt treatment (S6) | Alkali treatment (A15) |
| 4 | SA120091 | 88.1 | 71.1 | 4.4 | 0 |
| 5 | SA120093 | 52.5 | 18.3 | 5.33 | 1 |
| 6 | SA120097 | 89.6 | 64.8 | 16.8 | 2.6 |
| 8 | SA120745 | 46.8 | 13.5 | 20.3 | 0 |
| 11 | SA120826 | 46.8 | 42.6 | 17.6 | 0 |
| 16 | SA120850 | 71.7 | 86.8 | 19 | 1.67 |
| 25 | SA120161 | 56.6 | 43.4 | 4.16 | 0 |
| 66 | SA110522 | 61.2 | 21.4 | 2 | 1.2 |
| 67 | OA1410-1 | 60.2 | 12.5 | 25.6 | 0.16 |
| 76 | OA1413-7 | 60.6 | 4.3 | 2.3 | 0 |
| 79 | OA1414-2 | 54.8 | 27.9 | 4.4 | 0 |
| 80 | OA1414-3 | 88.6 | 82.5 | 8.6 | 0.5 |
| 83 | OA1414-6 | 42.5 | 35.4 | 5.3 | 0 |
| 105 | OA1426-4 | 83.3 | 81.5 | 13.5 | 0 |
| 108 | OA1426-7 | 87.2 | 74.3 | 4 | 0 |
| 112 | OA1429-1 | 91.7 | 53.0 | 21.2 | 0.83 |
| 115 | OA1430-1 | 80.0 | 71.0 | 19.8 | 0.3 |
| 118 | OA1432-2 | 88.1 | 85.3 | 6.33 | 0 |
| 119 | OA1432-3 | 72.7 | 61.2 | 21 | 0 |
| 121 | OA1432-5 | 78.8 | 64.6 | 17.7 | 0 |
| 125 | OA1433-1 | 84.3 | 70.0 | 4.2 | 0 |
| 126 | OA1434-1 | 95.5 | 84.7 | 16.3 | 0 |
| 127 | OA1435-1 | 75.4 | 77.7 | 9 | 0 |
| 128 | OA1435-2 | 88.2 | 92.7 | 5.8 | 2 |
| 132 | OA1438-1 | 82.6 | 59.1 | 12.3 | 0 |
| 133 | ND120042 | 81.4 | 68.6 | 9.7 | 1 |
| 137 | ND120430 | 51.9 | 23.7 | 6 | 0 |
| 140 | ND120609 | 93.4 | 67.2 | 11.6 | 0 |
| 153 | ND121147 | 84.0 | 56.8 | 7.83 | 0 |
| 155 | ND121165 | 25.5 | 16.3 | 13.4 | 1.2 |
| 165 | ND122569 | 82.9 | 73.2 | 15.7 | 0 |
| 168 | ND120494 | 35.0 | 3.4 | 12.3 | 0.17 |
| 170 | ND120497 | 19.2 | 28.4 | 8.7 | 8.0 |
| 171 | ND120580 | 55.2 | 4.8 | 3.3 | 0 |
| 176 | ND121383 | 54.1 | 16.5 | 17.3 | 1 |
| 183 | ND121722 | 46.5 | 24.6 | 3 | 1 |
| 184 | ND121726 | 55.9 | 28.4 | 2.3 | 0 |

**Continue Table 3**

|  |  | Germination rate (%) under | | Number of grains under | |
| --- | --- | --- | --- | --- | --- |
| Entry ID | Name | 22.5mmol.L^-1^ salt | 68.5mmol.L^-1^ alkali | Salt treatment (S6) | Alkali treatment (A15 |
| 214 | 09P05-DG | 95.8 | 52.4 | 9 | 0.6 |
| 21 | SA121014 | 42.7 | 40 | 4.7 | 0 |
| 219 | 09P06-ES | 41.5 | 3.3 | 5 | 0 |
| 227 | 09P09-EC | 48.4 | 25.4 | 8.3 | 0 |
| 233 | 09P09-FA | 50.0 | 17.7 | 10.7 | 0 |
| 240 | 09P09-GP | 37.9 | 26.7 | 9.7 | 0 |

Salt treatment S6: 150mmol.L^-1^ salinity 40L , Alkali treatment A15: 75mmol.L^-1^ salinity 40L ,for treatment details see Table 1.

The 43 genotypes were selected out of 248 genotypes for good or poor germination at a salt stress and an alkali stress (Experiment 2).

Combined results from Experiments 2 and 3.
