# Supplementary material for: Screening Oat Genotypes for Tolerance to Salinity and Alkalinity
Source: Front Plant Sci. 2018 Oct 2;9:1302. doi: 10.3389/fpls.2018.01302 (PMC6176118; doi:10.3389/fpls.2018.01302)
Supplement: Supplementary file 4 [file Table_4.doc]

| **Variety** | **Mean** | **Mean/LSD5%** | **Class5%** | **Variety** | **Mean** | **Mean/LSD5%** | **Class5%** |
| --- | --- | --- | --- | --- | --- | --- | --- |
| 210ND131936 | 3.676 | 1.96 | A | 144SA130502 | 1.295 | 0.69 |  |
| 8511W55-5 | 3.280 | 1.75 | A | 205ND132448 | 1.22 | 0.65 |  |
| 9111W64-27 | 2.433 | 1.3 | A | 19812QB03-BF | 1.208 | 0.65 |  |
| 9011W59-3 | 2.354 | 1.26 | A | 16510P10D-059B5 | 1.155 | 0.62 |  |
| 5411W10-7 | 2.345 | 1.25 | A | 28OA1455-1 | 1.148 | 0.61 |  |
| 203ND130776 | 1.920 | 1.03 | A | 16010P10C-008A5 | 1.090 | 0.58 |  |
| 5611W13-1 | 1.840 | 0.98 | A | 6711W03-17 | 1.088 | 0.58 |  |
| 103SA131566 | 1.780 | 0.95 |  | 119SA132045 | 1.083 | 0.58 |  |
| 199ND130775 | 1.547 | 0.83 |  | 24OA1451-1 | 1.080 | 0.58 |  |
| 6011W12-4 | 1.402 | 0.75 |  | 11OA1444-1 | 1.0744 | 0.57 |  |
| 5711W14-16 | 1.384 | 0.74 |  | 7411W37-7 | 1.033 | 0.55 |  |
| 5211W19-29 | 1.367 | 0.73 |  | 102SA131137 | 0.950 | 0.51 |  |
| 5111W55-17 | 1.299 | 0.69 |  |  |  |  |  |

**Table 4**  25 oat genotypes with the highest grain number among 262 genotypes under salt and alkali stresses in experiment 4

The values of grain number are based on the centering and scaling methods, they are supposed to be more accurate than the original data. Mean: the average value of grain number of each genotype. If the difference in Mean/LSD value between two genotypes is more than 1, then the difference in grain number between two genotypes is significant. Class5%: least significance at 5%.
