# Supplementary material for: Screening Oat Genotypes for Tolerance to Salinity and Alkalinity
Source: Front Plant Sci. 2018 Oct 2;9:1302. doi: 10.3389/fpls.2018.01302 (PMC6176118; doi:10.3389/fpls.2018.01302)
Supplement: Supplementary file 5 [file Table_5.doc]

**Table 5**  The germinating rates for 25 tolerant oat genotypes at mature stage under salinity(or alkalinity) in experiment 4

| **Variety** | **Salinity** | **Alkalinity** | **Variety** | **Salinity** | **Alkalinity** |
| --- | --- | --- | --- | --- | --- |
| 210ND131936 | 0.59 | 0.53 | 144SA130502 | 0.56 | 0.52 |
| 8511W55-5 | 0.51 | 0.41 | 205ND132448 | 0.62 | 0.43 |
| 9111W64-27 | 0.57 | 0.38 | 19812QB03-BF | 0.57 | 0.42 |
| 9011W59-3 | 0.62 | 0.57 | 16510P10D-059B5 | 0.49 | 0.52 |
| 5411W10-7 | 0.42 | 0.44 | 28OA1455-1 | 0.43 | 0.55 |
| 203ND130776 | 0.61 | 0.39 | 16010P10C-008A5 | 0.39 | 0.55 |
| 5611W13-1 | 0.45 | 0.52 | 6711W03-17 | 0.41 | 0.37 |
| 103SA131566 | 0.55 | 0.48 | 119SA132045 | 0.39 | 0.37 |
| 199ND130775 | 0.45 | 0.48 | 24OA1451-1 | 0.42 | 0.53 |
| 6011W12-4 | 0.49 | 0.42 | 11OA1444-1 | 0.51 | 0.50 |
| 5711W14-16 | 0.41 | 0.55 | 7411W37-7 | 0.43 | 0.37 |
| 5211W19-29 | 0.42 | 0.51 | 102SA131137 | 0.49 | 0.50 |
| 5111W55-17 | 0.52 | 0.50 |  |  |  |

Salinity: the germinating rate under salt median lethal concentration（LC50）; Alkalinity: the germinating rate under alkali LC50.
